# Intranasal prime-boost RNA vaccination elicits potent T cell response for lung cancer therapy

**DOI:** 10.1038/s41392-025-02191-1

**Published:** 2025-03-24

**Authors:** Hongjian Li, Yating Hu, Jingxuan Li, Jia He, Guocan Yu, Jiasheng Wang, Xin Lin

**Affiliations:** 1 Institute for Immunology and School of Basic Medical Sciences, Tsinghua University, Beijing, 10084 China; 2 College of Future Technology, Peking University, Beijing, 10084 China; 3 School of Pharmaceutical Sciences, Tsinghua University, Beijing, 10084 China; 4 Key Laboratory of Bioorganic Phosphorus Chemistry and Chemical Biology, Department of Chemistry, Tsinghua University, Beijing, 10084 China; 5 Changping Laboratory, Beijing, 10084 China; 6 Tsinghua-Peking Center for Life Sciences, Beijing, 10084 China

**Keywords:** Vaccines, Immunotherapy

## Abstract

The rapid success of RNA vaccines in preventing SARS-CoV-2 has sparked interest in their use for cancer immunotherapy. Although many cancers originate in mucosal tissues, current RNA cancer vaccines are mainly administered non-mucosally. Here, we developed a non-invasive intranasal cancer vaccine utilizing circular RNA encapsulated in lipid nanoparticles to induce localized mucosal immune responses. This strategy elicited potent anti-tumor T cell responses in preclinical lung cancer models while mitigating the systemic adverse effects commonly associated with intravenous RNA vaccination. Specifically, type 1 conventional dendritic cells were indispensable for T cell priming post-vaccination, with both alveolar macrophages and type 1 conventional dendritic cells boosting antigen-specific T cell responses in lung tissues. Moreover, the vaccination facilitated the expansion of both endogenous and adoptive transferred antigen-specific T cells, resulting in robust anti-tumor efficacy. Single-cell RNA sequencing revealed that the vaccination reprograms endogenous T cells, enhancing their cytotoxicity and inducing a memory-like phenotype. Additionally, the intranasal vaccine can modulate the response of CAR-T cells to augment therapeutic efficacy against tumor cells expressing specific tumor-associated antigens. Collectively, the intranasal RNA vaccine strategy represents a novel and promising approach for developing RNA vaccines targeting mucosal malignancies.

## Introduction

Cancer immunotherapy is a promising approach that leverages the immune system to target and eliminate cancer cells.^1^ Unlike conventional treatments such as chemotherapy and radiation, which directly kill tumor cells, immunotherapy enhances or restores the functionality of immune cells. Among various immunotherapeutic strategies, cancer vaccines have garnered significant attention.^2^ Cancer vaccines present tumor antigens to the antigen-presenting cells, inducing a tumor-specific T cell immune response. This strategy holds the potential for durable anti-tumor effects, addressing both tumor progression and recurrence. Tranditional vaccine carriers includes bacterial, virus, protein and tumor cells.^3^ A key challenge of traditional vaccines is effectively delivering tumor antigens and activating a robust immune response. Recent advancements in novel vaccine platforms, particularly RNA vaccines, have accelerated research in this field.^4^

RNA vaccines have emerged as a revolutionary immunization strategy, achieving remarkable success during the SARS-CoV-2 pandemic and underscoring their potential for rapid and effective disease prevention.^5,6^ Compared with conventional vaccine platforms, RNA vaccines offer a promising approach by enabling the expression of multiple target antigens while providing immunogenicity, thereby initiating robust antigen presentation and T cell responses.^7^ From the perspectives of drug production and clinical application, RNA vaccines present distinct advantages, including efficient large-scale production, higher purity, reduced impurities, established quality control protocols, and lower manufacturing costs.^8^ Currently, RNA-based vaccine platforms can be categorized into three main types: linear or linear modified RNA, circular RNA (circRNA), and self-amplifying RNA.^9^ While the approved RNA vaccines predominantly rely on linear modified RNA, their strict storage requirements highlight the need for improved stability to enable broader application. Self-amplifying RNA, despite its prolonged protein expression, faces challenges in production and storage due to its larger molecular size. In comparison, circRNA exhibits superior stability owing to its covalently closed ring structure, which confers enhanced thermostability and prolonged half-life in mammalian cells.^10^ Additionally, the production of circRNA is less complex, as it eliminates the need for nucleotide modifications, capping, and polyA tailing. Preclinical studies have demonstrated that circRNA vaccines, when administered via intramuscular injection, are effective against viral infections and subcutaneous tumors.^11,12^ However, further research is needed to explore alternative administration routes of circRNA vaccines to expand their therapeutic applications.

Traditional types of cancer vaccines are mainly designed to be administrated through intravenous injection.^13–17^ For RNA-based vaccines, the currently licensed SARS-CoV-2 vaccines are administered via intramuscular injection. Researchers have also explored intravenous options for delivering RNA to spleen tissues using optimized nanoparticles for cancer therapy.^18–21^ However, this approach necessitates systemic immune activation, which carries potential risks, as observed in adverse reactions reported for SARS-CoV-2 RNA vaccines.^22,23^ In contrast, non-invasive respiratory mucosal delivery methods, such as intranasal or inhalable routes, offer a promising alternative. These methods directly stimulate immune responses at the respiratory mucosal site,^24–26^ without the induction of systemic immune response. This strategy presents considerable potential for cancer immunotherapy, particularly for malignancies originating in mucosal tissues.^27^ Moreover, these non-invasive routes may mitigate the discomfort, risks, and complications linked to injections, such as needle-related injuries or infections.^28^ Consequently, the development of non-invasive RNA vaccines targeting mucosal tumors holds the potential to surpass current cancer vaccines in terms of efficacy and safety. Despite these advancements, the immunological mechanisms underlying antigen-specific T cell induction following RNA immunization, particularly via the mucosal route, remain poorly understood. A more comprehensive understanding of these processes could inform the rational design of more effective vaccines and combination therapies to enhance anti-tumor immune responses.

In this study, we utilized highly stable circRNA encapsulated in lung-targeting lipid nanoparticles (LNPs) to assess their potential as an intranasal cancer vaccine for treating lung cancers. Our findings demonstrate that the vaccine successfully induces protein expression in lung tissues with minimal adverse effects. This strategy also exhibited significant anti-tumor efficacy in lung cancer models, even in the presence of tumor cells expressing endogenous neoantigens. Mechanistically, the anti-tumor activity of the vaccine relies on conventional dendritic cells type 1 (cDC1s), which play a critical role in antigen-specific T cell priming. Both cDC1s and alveolar macrophages are responsible for boosting antigen-specific T cells in lung tissues. Moreover, the vaccine synergizes with adoptive T cell transfer therapy, augmenting tumor eradication by increasing the populations of both endogenous and transferred antigen-specific T cells. In addition, the vaccine can alter the endogenous T cell landscape by enhancing memory and effector phenotypes. Based on the unique properties of this intranasal vaccination strategy, we engineered vaccine-responsive CAR-T cells, designed to be enhanced by intranasal vaccination, for improved anti-tumor efficacy against tumor cells expressing tumor-associated antigens. Overall, this study provides a novel approach for treating mucosal malignancies through the non-invasive delivery of RNA vaccines.

## Results

### Intranasal RNA delivery via lipid nanoparticles initiates protein expression at lung tissues

Based on the previously published circRNA preparation strategy, the in-vitro transcription templates included a pair of exons and introns, CVB3 IRES, spacers, arms and the coding sequences.^29,30^ CircRNAs were obtained through the back-splicing reaction followed by purification through liquid chromatography. As circRNA is known to have better stability than mRNA, we first aimed to determine whether the circRNA vaccine exhibited superior thermostability compared to its linear counterpart when encapsulated in lipid nanoparticles (LNP). We utilized the clinically approved LNP formulation containing SM102, DSPC, DMG-PEG, and cholesterol. The RNAs were encoded with D2GFP, a destabilized GFP protein with a half-life of only two hours, to evaluate protein expression. Our results showed that circRNA exhibited higher protein expression levels than the linear RNA formulations when stored at both 4 °C and 37 °C, supporting the broader applicability and convenience of circRNA vaccines. (Supplementary Fig. 1).

**Fig. 1:**
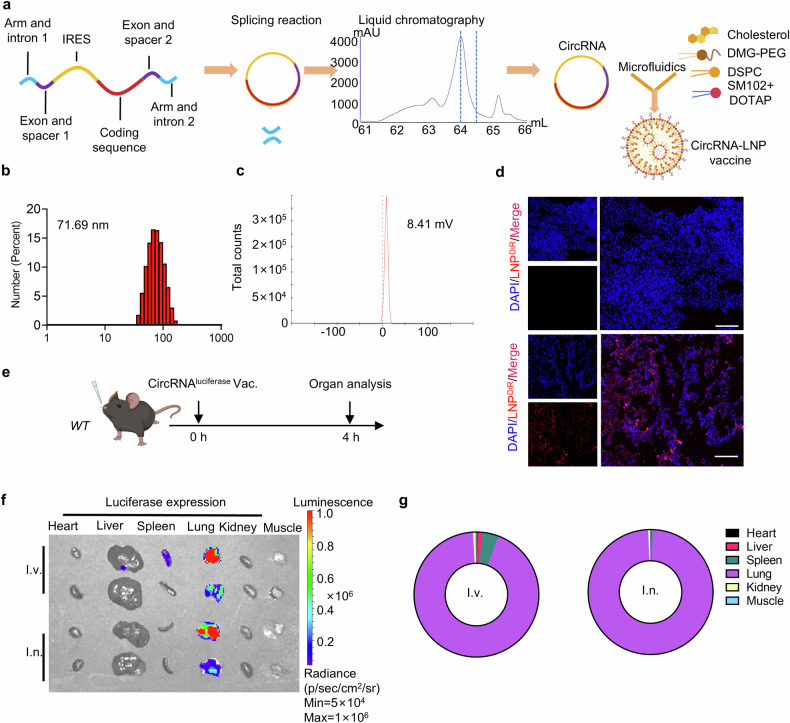
Intranasal circRNA delivery via lipid nanoparticles initiates protein expression at lung tissues. **a** Experimental diagram of the RNA-LNP vaccine manufacturing process. **b** Size distributions of the RNA-LNP vaccine. **c** Zeta potential of the RNA-LNP vaccine. **d** Representative dye-tracking fluorescent images of lung tissues. Mice were given with DiR-labeled LNPs via intranasal administration and lung tissues were isolated after 4 h. Scale bar, 100 μm. **e** Timeline of the experiment designed to evaluate the biodistribution of luciferase protein expression (2.5 μg circRNA per mouse). Imaging results (**f**) and statistical analysis (**g**) of luciferase protein expression among various organs. I.v., intravenous injection group. I.n., intranasal administration group

For intranasal delivery, we tested the efficiency of two clinically approved ionizable lipids (SM102 and ALC-0315) to form LNPs with DSPC, DMG-PEG and cholesterol. We characterized the size and zeta potential of two dye-containing LNPs (Supplementary Fig. 2a, b) and then we performed the in vivo assay. We observed that SM102-based LNP showed higher dye-tracking fluorescence intensity compared to ALC-0315 (Supplementary Fig. 2c, d). According to recent studies, the addition of cationic lipids, such as 1,2-dioleoyl-3-trimethylammonium-propane (DOTAP), can enhance lung-targeting efficiency after intravenous and intranasal administration.^31–33^ We optimized the delivery system by adding DOTAP to the SM102-based LNPs, resulting in a higher protein expression level (Supplementary Fig. 2e, f). Thus, this delivery strategy was used in the following experiments. The entire manufacturing process of circRNA vaccine is shown in Fig. 1a. Briefly, circRNA was prepared through in-vitro transcription and splicing reaction, followed by purification via liquid chromatography. Purified samples were mixed with five lipid components via microfluidics to form the circRNA-LNP complex. For the characterization of the RNA vaccine, the particle size was around 71.69 nm (Fig. 1b). The zeta potential was 8.41 mV (Fig. 1c). To monitor the biodistribution of the RNA vaccine, luciferase-coding circRNA and LNPs were mixed with DiR lipophilic dye. After intranasal administration, successful delivery was confirmed via dye-tracking fluorescent imaging (Fig. 1d). Furthermore, we aimed to compare the biodistribution of the RNA vaccine via different administration routes (Fig. 1e). We isolated vital organs 4 h after vaccination and found that the intranasal vaccine led to LNP accumulation and protein expression in lung tissues (Fig. 1f, g and supplementary Fig. 2g, h). However, the LNPs and protein expression could be detected in liver, lung, and spleen tissues after intravenous injection. Taken together, these data showed that the circRNA-LNP platform can initiate local protein expression at lung tissues after intranasal administration.

### Intranasal circRNA vaccine mediates potent anti-tumor response with limited adverse effects

**Fig. 2:**
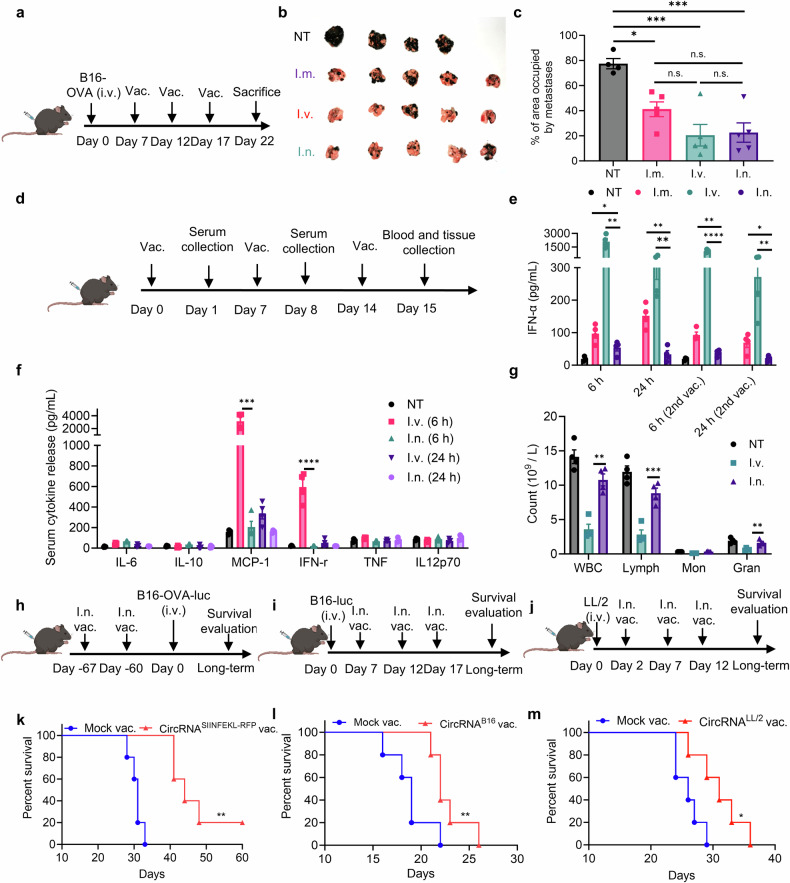
Intranasal circRNA vaccine mediates potent anti-tumor response with limited adverse effects. **a** Timeline of the experiment designed to evaluate the anti-tumor ability of different routes of circRNA vaccine. **b** Images of lung metastasis tumors at the endpoint in different groups. NT, not-treated group. I.m., intramuscular injection group. I.v., intravenous injection group. I.n., intranasal administration group. *n* = 4 for NT group. *n* = 5 for other groups. **c** The percentage of area occupied by metastatic tumor among various groups at the end point. Data were analyzed by one-way ANOVA with Tukey’s multiple comparisons test. **d** Timeline of the experiment designed to evaluate the systemic adverse effects of different routes of circRNA vaccine. (*n* = 4) **e**, **f** Serum cytokine release after administrated with the vaccine tested by mouse IFN-α Elisa kit (**e**) and mouse inflammation kit (**f**). Data were analyzed by Student’s t test to compare i.n. group with i.v. or i.m. group in e. Data were analyzed by one-way ANOVA with Tukey’s multiple comparisons test in f. **g** Blood routine analysis 24 h after the third vaccination. Data were analyzed by Student’s t test to compare i.n. group with i.v. group. **h**–**j** Timeline of the experiments to evaluate the anti-tumor ability of intranasal in various models. Mice received with LNP without RNA were considered as mock vaccine group. **h** Mice were immunized with SIINFEKL-coding circRNA twice, followed by rechallenging B16-OVA-luciferase cells 60 days after vaccination. **i** Mice were challenged with B16-luciferase cells and received three doses of B16-antigen-coding circRNA vaccine. **j** Mice were challenged with LL/2 cells and then received three doses of LL/2-antigen-coding circRNA vaccine. **k**–**m** Survival curves of the experiments related to **h**, **i** and **j**, respectively. *n* = 5 for each group. Data were analyzed via Kaplan-Meier analysis. For mice received with vaccination, each dose contained 2.5 μg circRNA. All data are represented as mean ± SEM

To systematically compare the anti-tumor efficiency of the circRNA vaccine administered through different routes, we established a lung metastasis B16 tumor model expressing the OVA antigen. Mice received SIINFEKL (class I (Kb)-restricted epitope of OVA antigen)-RFP-coding circRNA vaccines at specified time points (Fig. 2a). Compared to the untreated group, all three administration routes exhibited statistically comparable anti-tumor efficacy (Fig. 2b, c). However, the percentage of lung tissue occupied by metastatic foci appeared lower in the intranasal and intravenous groups compared to the intramuscular group. Then, we analyzed the acute toxicity of the circRNA vaccine administered through different routes. Previous studies have indicated that RNA vaccines can induce the secretion of pro-inflammatory cytokines, resulting in adverse effects.^34–36^ We first evaluated the IFN-α level in serum post-vaccination due to its key role in inflammation induction (Fig. 2d). Compared with intravenous and intramuscular injection groups, the serum levels of IFN-α were significantly lower in the intranasal groups (Fig. 2e). Then, we further analyzed other cytokines and similarly we found that intranasal groups showed lower cytokine level compared with intravenous injection. (Fig. 2f and supplementary Fig. 3). For the blood routine tests, it indicated a more significant reduction in blood and immune cells in the intravenous injection group compared to the intranasal group (Fig. 2g). To further verify the safety of intranasal administration, we monitored the body weight changes in mice throughout the treatment period (Supplementary Fig. 4). Although a slight decrease in body weight was observed in the vaccination groups, it was restored after a few days.

We further assessed the anti-tumor efficiency of the intranasal vaccine in different models (Fig. 2h–j). In prophylactic lung metastasis models, mice received two doses of the RNA vaccine, followed by a B16-OVA tumor cell challenge after 30 or 60 days. Bioluminescence images (Supplementary Fig. 5a, b) and survival curves (Fig. 2k and Supplementary Fig. 5c) showed that intranasal immunization inhibited tumor growth and significantly prolonged survival, suggesting the formation of memory T cells after vaccination. In therapeutic lung metastasis models, B16-luciferase and LL/2 cell lines without overexpressing OVA antigens were employed. Based on the published non-synonymous mutations of B16^37^ and LL/2^38^ cells, we designed B16-antigen-coding (Supplementary Fig. 5d–f) and LL/2-antigen-coding (Supplementary Fig. 6a, b) RNA vaccines, and mice received three doses of vaccination after the establishment of lung metastasis. In the B16 model, the survival curves showed that intranasal immunization significantly inhibited tumor growth and prolonged survival (Fig. 2l). In the LL/2 model, we observed that the immunized mice exhibited cytotoxicity with fewer tumor foci (Supplementary Fig. 6c, d) and more prolonged survival (Fig. 2m). In both models, we observed specific T cell activity targeting vaccine-encoded antigens in lung tissues (Supplementary Fig. 5f and 6e), highlighting the potential of intranasal circRNA for neoantigen vaccine applications. In summary, these data indicate that the intranasal circRNA vaccine elicits a potent anti-tumor response with fewer side effects compared to other routes of administration.

### The anti-tumor efficiency of intranasal circRNA vaccine is tumor antigen- and cDC1-dependent

**Fig. 3:**
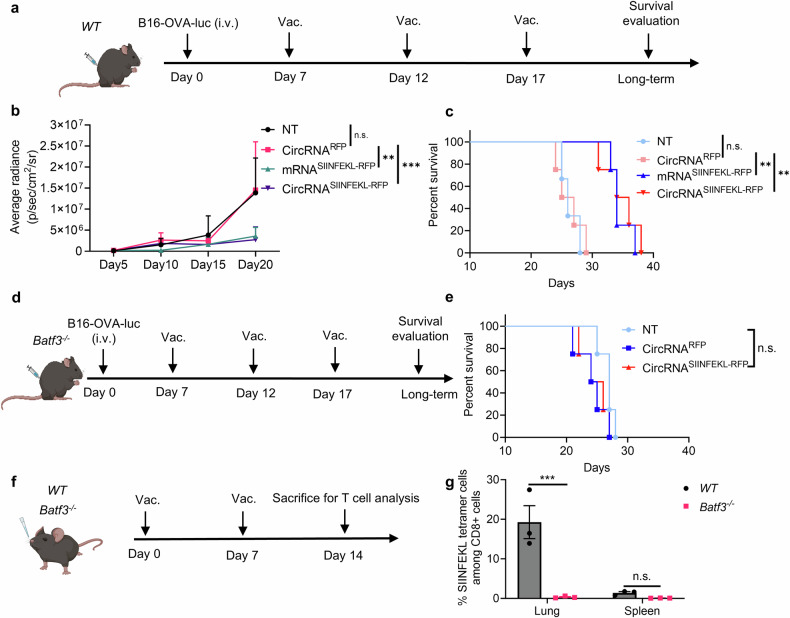
The anti-tumor ability of the intranasal circRNA vaccine is tumor antigen and cDC1 dependent. **a** Timeline of the experiment designed to evaluate the anti-tumor ability of the intranasal RNA vaccine. Wild-type (WT) mice were challenged with B16-OVA-luciferase cells and then left untreated (NT), treated with RFP-coding or SIINFEKL (OVA peptide)-RFP-coding RNA. Tumor growth was monitored through bioluminescence imaging. **b** Statistical analysis of the average bioluminescence radiance with different treatments. Data were analyzed with two-way ANOVA with Tukey’s multiple comparisons test. *n* = 3 for NT group. n = 4 for other groups. **c** Survival curve of the *WT* mice with different treatments. Data were analyzed via Kaplan-Meier analysis. **d** Timeline of the experiment designed to evaluate the anti-tumor ability of the intranasal RNA vaccine in *Batf3*^*−/−*^ mice. **e** Survival curve of the *Batf3*^*−/−*^ mice with different treatments. Data were analyzed via Kaplan-Meier analysis. *n* = 4 for each group. **f** Timeline of the experiment designed to evaluate the antigen-specific T cell induction after circRNA vaccination. *WT* or *Batf3*^*−/−*^ mice were intranasally immunized with SIINFEKL-RFP-coding circRNA followed by antigen-specific T cell evaluation at lung and spleen tissues (*n* = 3, repeated for three times). **g** Statistical analysis of the ratio of antigen-specific T cells. Data were analyzed with two-way ANOVA with Tukey’s multiple comparisons test. All data are represented as mean ± SEM

Inspired by the therapeutic potential of the intranasal vaccine, we aimed to further investigate the principles underlying its anti-tumor immune response. We first established a B16-OVA-luciferase lung metastasis model and confirmed the anti-tumor response (Fig. 3a). An RFP-coding RNA was used as an irrelevant antigen control. We also prepared linear mRNA and circRNA vaccines encoding SIINFEKL-RFP for vaccination. Bioluminescence image results (Fig. 3b and supplementary Fig. 7a) and survival curves (Fig. 3c) demonstrated that only tumor antigen-coding RNA vaccines could inhibit tumor cell growth, with no significant difference observed between mRNA and circRNA vaccines in this model. To further characterize the T cells induced by vaccination, we analyzed antigen-specific T cells in lung tissues following a two-dose vaccination regimen. Our results revealed an increase in both total antigen-specific T cells and CD103-positive antigen-specific T cell subset, confirming the successful induction of antigen-specific T cells (Supplementary Fig. 7b–e). Using an intravascular staining assay, we confirmed that the antigen-specific T cells induced by the intranasal vaccine primarily originate from the lung tissue rather than peripheral blood (Supplementary Fig. 7f–h). Furthermore, an in vivo CD8 depletion assay demonstrated that CD8 + T cells are indispensable for the vaccine’s anti-tumor efficacy (Supplementary Fig. 8a–d).

Given the preeminent role of CD8 T cells and vaccine-delivered antigens for initiating anti-tumor immunity, we aimed to test the influence of antigen presentation on anti-tumor ability. As cDC1 plays a major role in capturing and presenting antigens on MHC-I to promote CD8 + T cell immunity, we repeated the anti-tumor experiment in cDC1-knockout mice (Fig. 3d). Interestingly, the anti-tumor efficiency of the intranasal circRNA vaccine was diminished, suggesting that cDC1 is crucial for the vaccine-induced anti-tumor immune response (Fig. 3e). We further analyzed the antigen-specific CD8 + T cells induced by the vaccine (Fig. 3f). WT and *Batf3*^*−/−*^ mice received two doses of immunization, and T cells were detected in lung and spleen tissues. We found that cDC1 deficiency significantly decreased the level of antigen-specific CD8 + T cells at the lung tissue (Fig. 3g and supplementary Fig. 8e, f). To confirm that cDC1 presents the antigen after vaccination, we isolated the cDC1 from lung draining lymph node and coculture then with antigen-specific T cells (supplementary Fig. 8g). The activation of T cells following co-culture provides new evidence that cDC1 cells present antigens and play a key role in T cell induction. Thus, the expression of tumor antigen and the presence of cDC1 are indispensable for the anti-tumor T cell immune response induced by the intranasal circRNA vaccine.

### AMs and cDC1s are mainly responsible for boosting antigen-specific T cells in lung tissues after intranasal vaccination

**Fig. 4:**
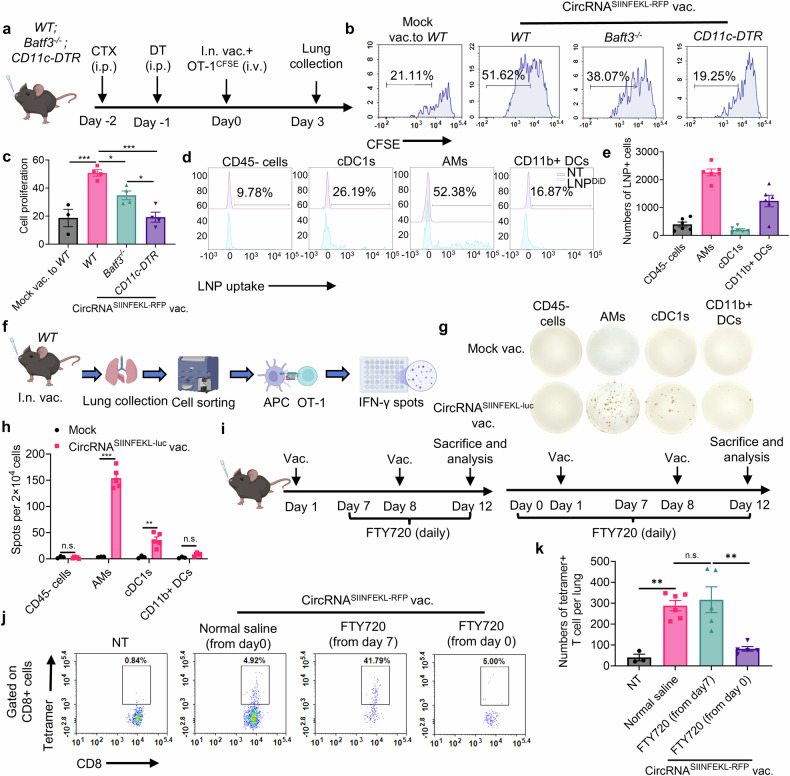
AMs and cDC1s are mainly responsible for boosting antigen-specific T cells in lung tissues after intranasal vaccination. **a** Timeline of the experiment to evaluate the role CD11c+ APCs on boosting antigen-specific T cells. *WT, Batf3*^*−/−*^ or *CD11c-DTR* mice were pre-treated with cyclophosphamide (CTX) and diphtheria toxin (DT) for lymphodepletion in all mice and CD11c+ cells depletion in CD11c-DTR mice. Activated OT-1 cells were labeled with CFSE and then transferred to mice immunized with intranasal circRNA vaccine. T cell proliferation at lung tissues was analyzed at day 3. **b**, **c** Representative flow cytometry histograms (**b**) and statistical results (**c**) of T cell proliferation with different treatments. Data were analyzed by one-way ANOVA with Tukey’s multiple comparisons test. *n* = 3 for mock vac. group; *n* = 4 for other groups. For LNP uptake analysis, mice received intranasal DiD-labeled LNP and lung tissues were analyzed 4 h later. **d**, **e** Representative flow cytometry histograms (**d**) and statistical results (**e**) of LNP uptake among different cell types. **f** Scheme of the experiment to test the potential APCs that can boost antigen-specific T cells. Mice were immunized with intranasal LNP without RNA (mock) or circRNA vaccine and 24 h later, lung tissues were collected and APCs were sorted and co-cultured with OT-1 cells for another 36 h in IFN-γ Elispot wells. (*n* = 3 for mock group; *n* = 5 for circRNA vaccine group, repeated for three times) Created with BioRender.com. **g**, **h** Representative images and statistical results of IFN-γ Elispot assay. Data were analyzed by Student’s t test. **i** Timeline of the experiment to explore whether vaccine can boost T cells at lung tissues. Mice were treated with FTY720 during the prime-boost process or only the boosting process or normal saline as the control group. The number of antigen-specific T cells at lung tissues was analyzed. **j**, **k** Representative plots (**j**) and statistical results (**k**) of the antigen-specific T cells with different treatments. Data were analyzed by one-way ANOVA with Tukey’s multiple comparisons test. All data are represented as mean ± SEM

Successful induction of robust and sustained T cell immunity requires not only the priming of T cells but also the boosting process through repeated vaccination. Therefore, we aimed to further explore the T cell boosting process following intranasal immunization. We employed WT mice, mice without cDC1, and mice without all CD11c+ cells to test their ability to boost activated antigen-specific T cells (Fig. 4a). Although depletion of cDC1 reduced T cell proliferation, the deficiency of CD11c+ cells completely blocked vaccine-induced T cell proliferation, suggesting that both cDC1 and other CD11c+ antigen-presenting cells are involved in boosting T cells (Fig. 4b, c and supplementary Fig. 9a). To identify the cell types that support T cell proliferation, LNPs were labeled with DiD lipophilic dye and intranasally administered to mice. We then stained the major populations of CD11c+ cells at lung tissues, including alveolar macrophages (AMs), cDC1s, CD11b+ DCs, and CD11c- CD45- cells as the control group. We found that LNPs were predominantly engulfed by AMs, with other cells also capable of engulfing LNPs but in smaller numbers (Fig. 4d, e, supplementary Fig. 9b–d). Moreover, the production of IFN-γ was augmented when isolated AMs and cDC1s were co-cultured with OT-1 cells, while there was no significant difference in the CD11b+ or CD45- groups (Fig. 4f–h). We also performed immunofluorescence (IF) staining of lung tissues to investigate the interactions between antigen-presenting cells (APCs) and antigen-specific T cells (Supplementary Fig. 10). In the vaccination group, we observed interactions between antigen-specific T cells and CD11c+ cells, as well as between antigen-specific T cells and AMs. These findings provide new evidence that both AMs and dendritic cells (DCs) contribute to the activation and enhancement of antigen-specific T cells. We next assessed whether the intranasal vaccine primed and boosted T cells directly in lung tissues. To do this, FTY720 was employed to block T-cell egress from lymphoid tissues. Mice were treated with FTY720 daily starting before the priming or boosting process, and antigen-specific T cells were detected in lung tissues at the endpoint (Fig. 4i). During the boosting process, FTY720 treatment had no impact on the number of antigen-specific T cells in lung tissues, indicating that the vaccine boost led to antigen presentation and T cell proliferation within the local lung tissues (Fig. 4j–k and supplementary Fig. 9d). However, the number of antigen-specific T cells in lung tissues significantly decreased (Fig. 4k), with no impact on the mediastinal lymph node when mice were treated with FTY720 starting before the first immunization (Supplementary Fig. 11). It suggested that the initial priming of antigen-specific T cells occurred outside lung tissues, relying on draining lymph nodes. Thus, these data indicate that the intranasal circRNA vaccine can boost T cells directly in lung tissues through the actions of AMs and cDC1s.

### Intranasal circRNA vaccine boosts both endogenous and transferred antigen-specific T cells to promote anti-tumor immune response

**Fig. 5:**
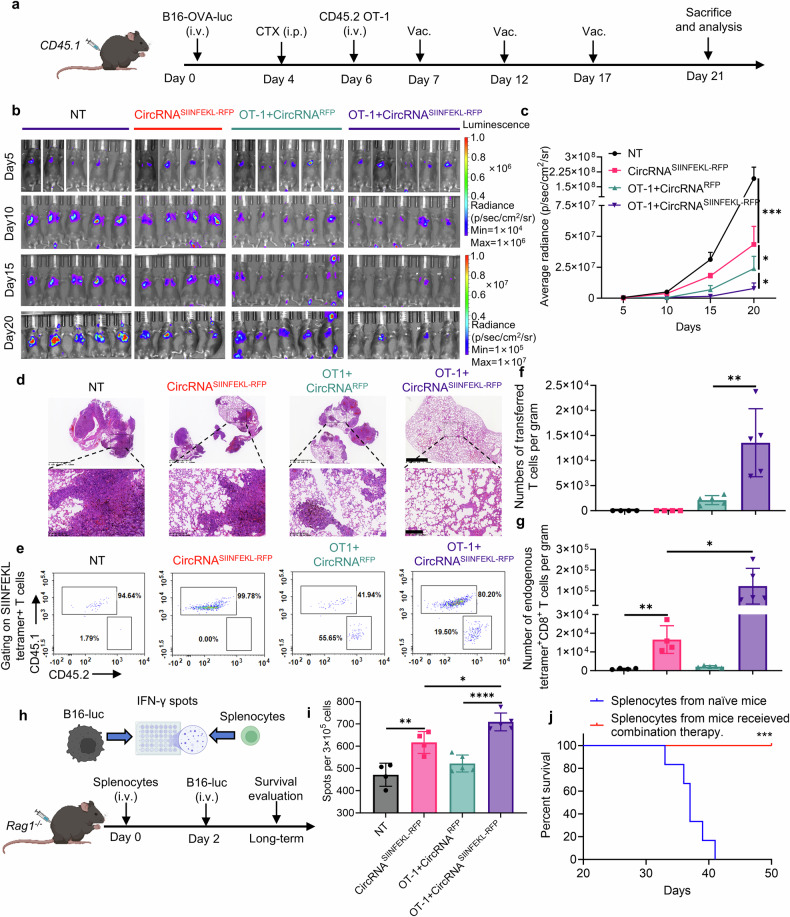
Intranasal circRNA vaccine boosts adoptive transferred antigen-specific T cells for enhanced anti-tumor immune response. **a** Timeline of the experiment to evaluate the anti-tumor ability of combination therapy with intranasal circRNA vaccine and transferred OT-1 cells. **b**, **c** Bioluminescence images (**b**) and statistical result of average radiance (**c**) at different time points. Data were analyzed with two-way ANOVA with Tukey’s multiple comparisons test. **d** Representative images of HE staining to evaluate the tumor formation at day 21. Scare bar, 1250 μm (upper) or 200 μm (lower). **e** Representative plots of the transferred and endogenous antigen-specific T cells among different groups. **f**, **g** Statistical analysis of antigen-specific T cells in **e**. Data were analyzed by Student’s t test. **h** Scheme of the experiments to evaluate the role of endogenous T cells in killing antigen-loss tumor cells. Splenocytes were co-cultured with B16-luciferase cells (without OVA antigen) for IFN-γ Elispot assay. Splenocytes from the mice in the combination therapy group were transferred to *Rag1*^*−/−*^ followed by B16-luciferase cells challenge. Created with BioRender.com. **i** Statistical results of the number of IFN-γ spots. Data were analyzed by one-way ANOVA with Tukey’s multiple comparisons test. **j** Survival curve of the *Rag1*^*−/−*^ mice, *n* = 6 for each group. Data were analyzed via Kaplan-Meier analysis. All data are represented as mean ± SEM

Based on the above findings, we hypothesized that intranasal RNA vaccine may synergize with transferred antigen-specific T cells for enhanced anti-tumor ability. We conducted experiments using a *WT* mouse model. Mice were initially challenged with B16-OVA-luciferase tumor cells and underwent lymphodepletion with CTX before receiving treatment with circRNA vaccine, adoptive cell therapy (ACT), or a combination of both therapies (Fig. 5a). Bioluminescence imaging and histopathological examination (HE staining) demonstrated that while vaccine alone or OT-1 cells with mock vaccine could inhibit tumor growth, the combination therapy resulted in the most pronounced tumor control (Fig. 5b–d). We also quantified antigen-specific T cell numbers for both endogenous and transferred T cells, revealing that the vaccine effectively expanded both populations (Fig. 5e–g).

Given the potential secondary effects of the vaccine-induced T cell response, it is possible that immune responses against secondary antigens, distinct from the original vaccine target, could be triggered. In this study, the combination of vaccination and ACT induces T cell-mediated destruction of tumor cells expressing the OVA antigen. This destruction releases additional tumor antigens, which may be captured by antigen-presenting cells, subsequently leading to the activation of endogenous T cells targeting OVA antigen-loss tumors. This antigen-spreading phenomenon has been observed in both preclinical and clinical studies involving immunotherapy.^39^ Thus, we assessed the anti-tumor capability of splenocytes from vaccinated mice when co-cultured with B16 cells lacking the OVA antigen (Fig. 5h). Remarkably, splenocytes from immunized mice exhibited increased production of IFN-γ, indicating that the vaccine can induce antigen spreading to target antigen-loss tumor cells (Fig. 5i). Moreover, transferred splenocytes from the mice in the combination therapy group prevented tumor formation upon rechallenge with B16 cells lacking the OVA antigen (Fig. 5h, j), suggesting that the combination therapy may facilitate the generation of memory T cells capable of targeting tumor cells through alternative antigens. These findings underscore the potential of combining intranasal RNA vaccine with ACT to enhance anti-tumor immunity by expanding both endogenous and adoptively transferred T cells, inducing antigen spreading, and potentially fostering the formation of memory T cells against tumor cells expressing other antigens.

### Intranasal circRNA vaccine induces functional and phenotypic changes in endogenous T cells

**Fig. 6:**
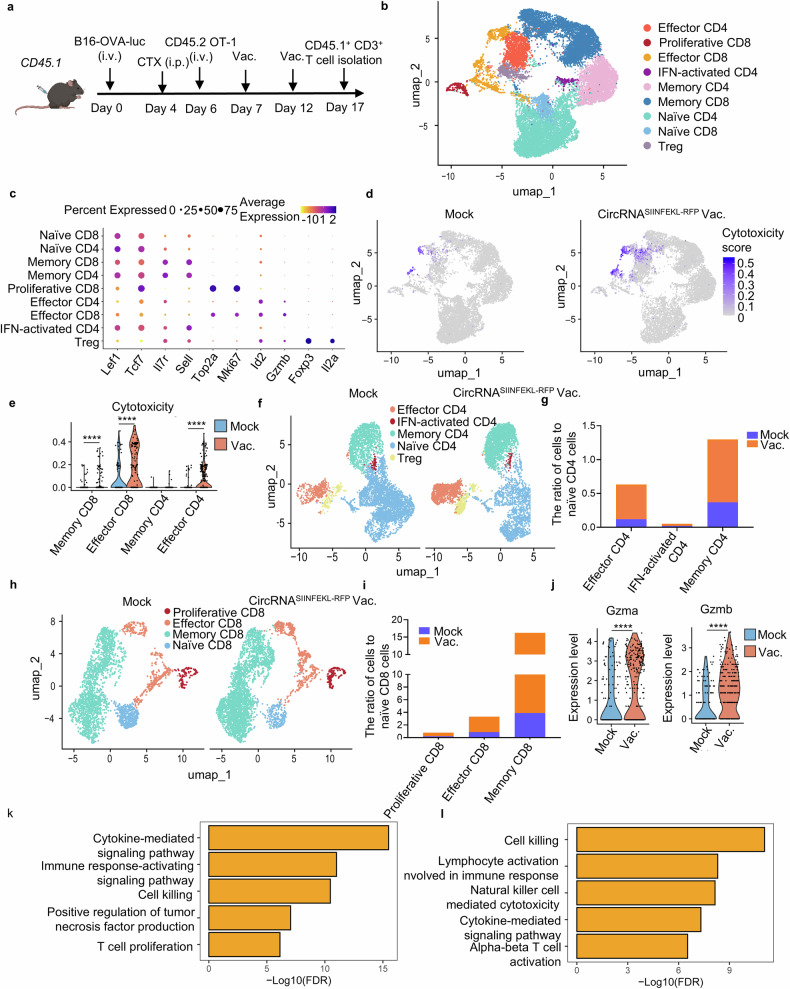
Endogenous T cells show transcriptional changes with enhanced cytotoxic function and more memory-like phenotype in response to intranasal vaccine. **a** Timeline of the experiment to isolate the endogenous T cells for single-cell RNA sequencing. Mice received LNP without RNA (mock, *n* = 3) or SIINFEKL-RFP-coding circRNA vaccine group (vac., *n* = 3) for two doses. Cells from lung tissues were mixed for staining and sorting in the same group. Sorted cells were sent for single-cell RNA sequencing. **b** UMAP plot of CD3 + T cells colored by clusters. **c** Dot plots showing differential expression of marker genes in endogenous CD3 + T cells. **d** UMAP plot of the T cells with cytotoxic function in mock group (mock) and vaccine group (vac.). **e** Statistical analysis of the cytotoxic gene expression in different clusters. Data were analyzed through the Wilcoxon signed-rank test. **f** UMPA plot of CD4 + T cells in two groups. **g** Statistical analysis of the CD4+ cell number of various clusters compared to their naïve cells in mock and vac group. **h** UMAP plot of CD8 + T cells in two groups. **i** Statistical analysis of the CD8+ cell number of various clusters compared to their naïve cells in mock and vac group. **j** Statistical analysis of *Gzma* and *Gzmb* expression in effector CD8 clusters. Data were analyzed through the Wilcoxon signed-rank test. **k**, **l** GO enrichment analysis showing the enriched terms of vac. group compared with mock group in effector CD4 (**k**) and effector CD8 (**l**) clusters

Building on our previous findings, we aimed to comprehensively examine alterations in endogenous T cells using single-cell RNA-seq analysis of CD3+ cells from lung tissues in tumor-bearing mouse models (Fig. 6a). Unsupervised clustering of the transcriptome data identified nine major T cell subsets: naïve CD4 and CD8 cells (marked by *Lef1* and *Tcf7* expression with relatively low *Il7r* and *Sell* levels), memory CD4 and CD8 cells (expressing *Il7r* and *Sell*), proliferative CD8 cells (characterized by *Top2a* and *Mki67* expression), effector CD4 and CD8 cells (with *Id2* and *Gzmb* expression), Treg cells (featuring *Foxp3* and *Il2ra*), and IFN-activated CD4 T cells (marked by *Ifit1*, *Ifit3*, and *Isg15* expression) (Fig. 6b, c). We specifically identified a cytotoxic T cell cluster characterized by a signature of cytotoxicity-associated genes (*Gzma, Gzmb, Gzmc, Gzmd, Gzme, Gzmf, Gzmg, Gzmk, Gzmm*), which showed significantly heightened cytotoxic activity in the vaccine group compared to controls (Fig. 6d). Further analysis revealed that cytotoxic capabilities were notably upregulated in memory and effector T cell clusters within the vaccinated group (Fig. 6e). Additionally, we analyzed the abundance and relative proportions of each T cell cluster within CD4 and CD8 populations separately, finding increased numbers and ratios of effector and memory cells in the vaccine group (Fig. 6f–i). Importantly, the anti-tumor function of effector CD8 T cells were significantly enhanced in the vaccine group, as evidenced by elevated expression levels of *Gzma* and *Gzmb* (Fig. 6j). Gene Ontology (GO) enrichment analysis further indicated that pathways related to T cell function and anti-tumor cytotoxicity in CD4 and CD8 effector cells were enriched in the vaccine group (Fig. 6k, l). We validated the functionality of both endogenous and transferred T cells through intracellular IFN-γ staining (Supplementary Fig. 12). An increased level of IFN-γ was observed in both populations, indicating enhanced cytotoxic activity of the T cells upon encountering tumor cells. Overall, this comprehensive analysis demonstrates that the intranasal circRNA vaccine drives substantial changes in the landscape of endogenous T cells, enhancing their cytotoxicity and memory functions and thereby bolstering the anti-tumor immune response.

### Intranasal circRNA vaccine augments the anti-tumor efficacy of CAR-T cell therapy against tumor cells expressing specific tumor-associated antigens

**Fig. 7:**
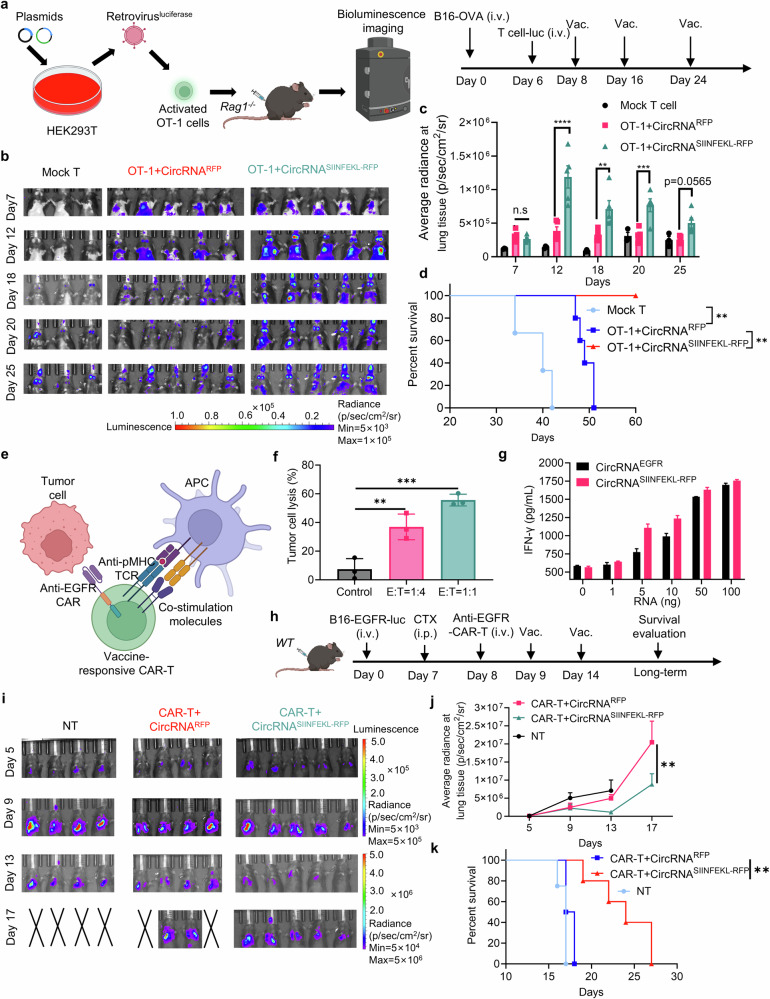
Intranasal circRNA vaccine augments the anti-tumor efficacy of CAR-T cell therapy against tumor cells expressing specific tumor-associated antigens. **a** Scheme of the experiment to track the migration and expansion of transferred OT-1 cells. Cells were activated, infected with luciferase-coding retrovirus and then transferred to tumor-bearing *Rag1*^*−/−*^ mice. T cell proliferation at lung tissues was monitored by bioluminescence imaging. Created with BioRender.com. **b** Bioluminescence images of the mice at different time points. Mock T cells were cells isolated from *WT* mice, circRNA^RFP^ was used as an irrelevant vaccine control. **c** Statistical results of average radiance at lung tissues at different time points. Data were analyzed with two-way ANOVA with Tukey’s multiple comparisons test. **d** Survival curve of the mice after different treatments. Data were analyzed via Kaplan-Meier analysis. **e** Scheme of the vaccine-responsive CAR-T cell design. OT-1 cells were activated and infected with anti-EGFR CAR-coding retrovirus. The CAR-T cells can kill EGFR-expressing tumor cells through their CAR molecule and can be stimulated with SIINFEKL antigen-loaded APCs through their TCR-machinery. Created with BioRender.com. **f** Statistical analysis of tumor cell lysis after coculture with EGFR-expressing B16 cells and the CAR-T cells at different E:T ratio. Data were analyzed by one-way ANOVA with Tukey’s multiple comparisons test. **g** Analysis of IFN-γ secretion after coculture with the vaccine-responsive CAR-T cells and DC2,4 cells. DC2.4 cells were pre-transfected with different doses of RNA 24 h before coculture. **h** Timeline of the experiment to evaluate the anti-tumor ability of combination therapy with intranasal RNA vaccine and transferred CAR-T cells. **i**, **j** Bioluminescence images (**i**) and statistical result of average radiance (**j**) at different time points. Data were analyzed with two-way ANOVA with Tukey’s multiple comparisons test. (**k**) Survival curve of the mice with different treatments. Data were analyzed via Kaplan-Meier analysis. All data are represented as mean ± SEM

In addition to examining the effects of the vaccine on endogenous T cells, we also investigated its direct impact on adoptive transferred T cells. To visualize the dynamic biodistribution and long-term performance of vaccine-boosted transferred T cells during the anti-tumor process, we used B16-OVA bearing *Rag1*^*−/−*^ mice injected with OT-1 cells co-expressing luciferase, followed by immunization with the intranasal circRNA vaccine (Fig. 7a). In vivo bioluminescence imaging revealed that the vaccine drove T cell expansion in lung tissues, and repeated doses sustained T cell numbers at a relatively high level compared to the mock vaccine group (Fig. 7b, c). Assessing the anti-tumor efficiency of various treatments, we observed that intranasal vaccination significantly prolonged the survival of T cell-transferred mice (Fig. 7d). These findings illustrate that intranasal RNA vaccine boosting enables transferred antigen-specific T cells to expand in lung tissues, thereby enhancing their efficiency in lung cancer treatment.

To broaden the application of intranasal RNA vaccine, we leveraged their capacity to boost antigen-specific T cells. In doing so, we developed a vaccine-responsive CAR-T cell strategy that not only responds to the vaccination but also targets tumor cells expressing specific surface tumor-associated antigens. These antigens are typically challenging for conventional cancer vaccine approaches which are limited to target MHC-presented immunogenic peptides. This approach aims to eliminate tumor cells via the CAR molecule while simultaneously promoting in-situ boosting of immune responses through the TCR machinery (Fig. 7e). The CAR-T cells showed the ability to kill EGFR-overexpressing B16 tumor cells at different E:T ratio in vitro (Fig. 7f). We then examined the activation of CAR-T cells through their CAR or TCR. DCs were pre-transfected with varying doses of EGFR- or SIINFEKL-RFP-coding circRNA, and CAR-T cells were co-cultured with these DCs, followed by T cell activation and cytokine analysis. The secretion of IFN-γ increased (Fig. 7g), and the expression of CD25 and CD69 was upregulated as the RNA dose increased (Supplementary Fig. 13). These data demonstrated the CAR-T cells could exhibit specific function after stimulation through both EGFR-CAR and predefined TCR. Subsequently, an EGFR-expressing B16 lung metastasis tumor model was established in *WT* mice. CAR-T cells were then transferred to these mice, followed by the administration of an intranasal circRNA vaccine (Fig. 7h). Bioluminescence imaging (Fig. 7i), statistical analysis, and survival curves (Fig. 7j, k) indicated that CAR-T cell therapy combined with a mock vaccine initiated only a modest anti-tumor response. In contrast, CAR-T cell therapy paired with a SIINFEKL-encoding vaccine significantly inhibited tumor growth and prolonged survival. These findings suggest that intranasal RNA vaccines are effective for in-situ boosting of CAR-T cells to target and eliminate tumor cells expressing specific surface antigens.

## Discussion

A significant challenge in developing effective cancer vaccines lies in eliciting potent T cell immune responses, which are often hindered by insufficient antigen uptake and presentation.^2^ RNA vaccines offer the ability to deliver desired antigens and effectively activate antigen-presenting cells for T cell induction, which is a novel perspective for cancer treatment.^4^ However, existing studies predominantly focus on intravenous RNA vaccines, which can induce systemic inflammatory responses to boost innate immunity and reprogram the tumor microenvironment (TME) for enhanced anti-tumor T cell responses.^18–21^ Despite their efficiency, these systemic approaches may pose risks of adverse effects, especially in patients with pre-existing inflammation.^34–36^ To address these challenges, we propose novel vaccine strategies that preserve anti-tumor efficiency while minimizing systemic inflammation, particularly through mucosal vaccination for mucosal malignancies. In our study, we provided a strategy of intranasal circRNA vaccination for lung cancer treatment. This strategy activates local immune responses and effectively suppresses tumor progression with minimal systemic inflammation. Previously, orthotopic head and neck or lung cancers were reported to be inhibited by a peptide-based cancer vaccine via intranasal mucosal but not the intramuscular rout.^40,41^ Interestingly, although our findings show that intranasal and intravenous injections of the circRNA vaccine appear better tumor control compared with intramuscular injection, the intramuscular vaccine also inhibit tumor growth. This discrepancy from previous studies may be attributed to differing levels of systemic T cell activation induced by distinct vaccine formulations following injection administration. Taken together, these results all suggest that the noninvasive intranasal immunization is indeed beneficial for local lung cancer treatment with minimal systemic inflammation.

In cancer vaccine development, tumor antigen-specific T cells are pivotal for effective anti-tumor immunity. However, the mechanisms underlying T cell priming and boosting, particularly with RNA vaccines, remain poorly understood. A study on the Pfizer-BioNTech SARS-COV2 mRNA vaccine has highlighted the role of cDC1 in T-cell induction.^42^ Similarly, our research underscores the critical role of cDC1 in mediating the anti-tumor effects of RNA vaccines, demonstrating diminished CD8 T cell responses in cDC1-deficient mice. While the priming phase involves specific DC subtypes, boosting antigen-specific T cells may engage additional cell types. For instance, alveolar macrophages (AMs) were found to be essentially for CD8 T cell expansion following intranasal protein vaccination.^43^ In our current data, both AMs and cDC1 can boost antigen-specific CD8 T cells after intranasal vaccination. This is not entirely consistent with the previous study, indicating that different vaccine approaches modulate host T-cell response with distinct antigen-presentation principles. Moreover, antigen expression duration may influence vaccine-induced immune responses differently. For instance, circRNA vaccines encoding viral antigens have been shown to induce higher humoral immune responses compared to conventional mRNA vaccines.^11^ In our previous^12^ and current studies, tumor antigen-coding mRNA and circRNA reveal similar anti-tumor efficiency, aligning with another comparative analysis of mRNA and self-amplifying RNA with longer protein expression duration.^44^ They showed self-amplifying RNA and mRNA drove anti-tumor response with few differences. These results indicate that prolonged antigen expression may favor humoral immunity with minimal impact on CD8 + T cell response. Nevertheless, compared with mRNA, the circRNA vaccine retains its unique advantage in terms of stability, especially when storage at 4 °C as showed in our data and a previous study,^11^ which supported the broaden applicability and convenience of circRNA vaccines.

In general, cancer vaccines are mainly designed to stimulate endogenous T cells to recognize and attack tumor cells through tumor-specific antigens or neoantigens presented onto MHC-I molecules. This process, although effective, typically requires time to establish durable immunity. In contrast, adoptive T cell therapies, such as TCR-T and CAR-T therapies, use ex vivo activated T cells to target MHC-peptide complexes or surface tumor-associated antigens, enabling a direct and rapid therapeutic response. However, these approaches often face challenges in achieving sustained long-term persistence, particularly in solid tumors. These limitations suggest the potential for synergistic integration with vaccines and adoptive T cell therapies to enhance both efficacy and durability of immune responses. Previous studies have shown that intravenous mRNA vaccines or subcutaneously peptide vaccines enhance the function of transferred CAR-T and TCR-T cells,^18,45,46^ echoing our findings of intranasal circRNA promoting the accumulation and expansion of transferred T cells in lung tissues, thus prolonging survival in combination therapy. In addition, we designed a combination treatment strategy in a tumor-associate-antigen model with CAR-T cells and the intranasal vaccine. The approach provides two significant advantages, enhancing CAR-T cell persistence and enabling redirection of T cells to target tumor-associated antigens independent of MHC presentation on tumor cells.

It is well-established that antigens delivered by vaccines can elicit antigen-specific T-cell responses. Additionally, vaccine-boosted T cells and the vaccines themselves can interact with the host immune system. A study has demonstrated that vaccines can enhance the efficiency of host T cells in treating tumors with antigen heterogeneity.^45^ We have observed a similar phenomenon in our study. Splenocytes from vaccinated mice exhibited increased cytotoxicity against OVA antigen-loss tumor cells and maintained memory responses that protected mice from rechallenging with antigen-loss tumors. This antigen-spreading phenotype may enhance therapeutic efficiency in clinical settings when treating tumors with antigen-loss or antigenic heterogeneity. Another study suggests that intranasal virus delivery can reshape the local immune microenvironment and enhance the cytotoxic function of T cells.^47^ Similarly, our data revealed transcriptional changes in endogenous T cells, characterized by enhanced cytotoxic abilities and an increased number of memory T cells in lung tissues.

**Fig. 8:**
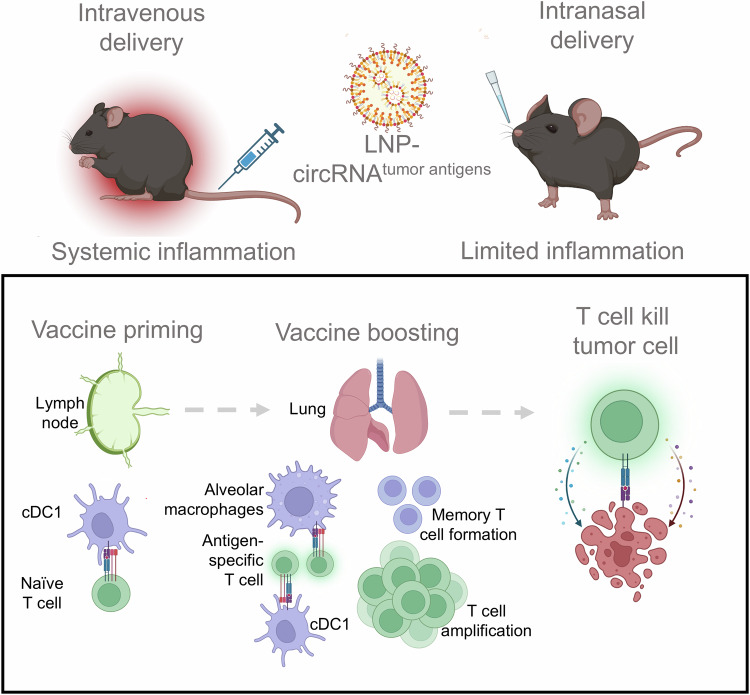
Proposed model of the intranasal circRNA vaccine for cancer therapy. Compared with conventional intravenous vaccine, intranasal delivery of circRNA vaccine results in fewer systemic adverse effects. For the mechanism of action of the vaccine, cDC1s are responsible for activating T cells at lymph nodes in the priming phase. Then, both cDC1s and AMs play significant roles in amplifying pre-existing endogenous or adoptive transferred antigen-specific T cells directly within lung tissues. Moreover, the priming-boosting vaccination strategy enhances the generation of memory T cells, which are critical for long-term immune surveillance and response. Consequently, the endogenous or adoptive transferred T cells trained by the vaccine are capable of initiating a robust and targeted anti-tumor immune response. Overall, the intranasal delivery of the circRNA vaccine leverages localized immune activation and amplification, presenting a promising approach for cancer immunotherapy with minimized systemic adverse effects. Created with BioRender.com

In summary, our study demonstrates that intranasal circRNA vaccines can induce potent T-cell immune responses against lung cancers while minimizing systemic inflammation. We elucidate the roles of cDC1 and AMs in T cell priming and boosting processes and illustrate that the vaccine strategy synergizes effectively with cell therapy, enhancing anti-tumor efficiency (Fig. 8). Our findings contribute to the rational design and optimization of mucosal cancer vaccines, offering a novel approach to achieving robust local anti-tumor activity with limited systemic effects.

## Materials and Methods

### Animals and cell lines

Female C57BL/6 J mice (6-8-week-old) and CD45.1 C57BL/6 J mice (6–8-week-old) were purchased from the laboratory animal resources center of Tsinghua University. Female Balb/c mice (6-week-old) were purchased from Beijing Vital River Laboratory Animal Technology. *OT-1* (JAX stock no. 003831) mice were kindly provided by Chen Dong’s lab (Tsinghua University). *Batf3*^*−/−*^(JAX stock no.013755) mice were kindly provided by Yangxin Fu’s lab (Tsinghua University). *CD11c-DTR* (JAX stock no. 004509) and *Rag1*^*−/−*^(JAX stock no. 002216) mice were purchased from Jackson Laboratory. All mice were kept under SPF-grade conditions in the animal facility of Tsinghua University. All the mouse experiments were strictly adhered to the compliance standards of the Institutional Animal Care and Use Committee of Tsinghua University. HEK293T, B16 and LL/2 cell lines were purchased from ATCC. B16-OVA cells were kindly provided by Meng Xu’s lab (Tsinghua University). B16-luciferase and DC2.4 cells were kindly provided by Rui Kuai’s lab (Tsinghua University). All cells were cultured in DMEM medium (with 10% FBS and 1% penicillin-streptomycin solution) at 37 °C and 5% CO2.

### Plasmid

For in-vitro transcription (IVT) backbone, the plasmid template contains T7 promoter, homology arm, elements from permuted intron-exon (PIE) construct, spacer, CVB3 IRES, and coding sequence. The luciferase coding sequence was cloned into a retroviral vector for virus packaging. The extracellular domain of human EGFR, transmembrane domain and luciferase coding sequence was cloned into a retroviral vector for virus packaging. For CAR-T generation, the construct contains GM-CSF signal peptide, the scfv of cetuximab, CD28 transmembrane and intracellular domain, CD3 intracellular domain, IRES and RFP coding sequence. All the sequences are attached at Supplementary Table 1.

### Preparation of RNA

The IVT templates were amplified by PCR (PrimeSTAR Max DNA Polymerase, Takara), followed by agarose electrophoresis and gel extraction (GeneJET Gel Extraction Kit, Thermo). CircRNA was manufactured as before.^12^ Briefly, IVT reaction was performed by using the T7 High Yield RNA Transcription Kit (Syngenebio). RNA was purified by DNase I digestion and lithium chloride precipitation. To circularize RNA, the reaction was performed at 55 °C for 15 min in T4 RNA Ligase Reaction Buffer (NEB) and 2 mM GTP (ApexBio). To remove byproducts, RNA was further purified via liquid chromatography using a 7.8 × 300 mm size-exclusion column with a particle size of 5 μm and pore size of 2000 Å (Sepax Technologies, 215980-7830). Enriched circRNA was subsequently precipitated by ammonium acetate and stored at −80 °C. Linear mRNA transcription and capping were performed by using the T7 Co-transcription RNA Synthesis Kit (Syngenebio) and uridine was replaced by N1-methylpseudouridine (m1Ψ). Products were purified via DNase I digestion, lithium chloride precipitation, followed by polyA-tailing via the E. coli Poly (A) Polymerase (Novoprotein). Products were precipitated by ammonium acetate and stored at −80 °C. All the RNA vaccines used in the study are attached at Supplementary Table 2.

### Preparation of lipid nanoparticle (LNP)

The ionizable lipid SM102 and ALC-0135 were purchased from Sinopeg. DOTAP, DMG-PEG 2000, cholesterol, and DSPC were purchased from MCE. Unless specifically mentioned in the text, the ethanol phase of lipid nanoparticle contains SM102, DOTAP, DSPC, cholesterol and DMG-PEG 2000 at the molar ratio of 40:10:10:38:2. RNA was dissolved in an aqueous phase composed of 100 mM citrate buffer (pH = 4). The RNA: lipid mass ratio was 1:30. LNPs were prepared by mixing the aqueous phase and ethanol phase at a 3:1 ratio via microfluidic mixing devices (Micro&Nano Technologies). Obtained LNPs were dialyzed against PBS for 4 h (MWCO = 3.5 kDa, Thermo). For LNP characterization, 50 μl samples were resuspended in 950 μl H_2_O and analyzed using dynamic light scattering (DLS) performed on a Zetasizer Nano (Malvern Instruments, Malvern, UK).

### Biodistribution of LNP

To monitor the uptake and biodistribution of LNP in vivo, DiR or DiD were added to the ethanol phase to encapsulate luciferase-coding circRNA for IVIS or flow cytometry, respectively. The DiR/DiD: lipid mass ratio was 1:1000. LNPs were filtered through a 0.22 μm filter and then intranasally administrated into C57BL/6 J mice pre-treated with isoflurane (2.5 μg RNA in 30 μl per mouse). After 4 h, mice were injected intraperitoneally with D-luciferin (150 mg/kg, Yeasen) and sacrificed 5 min later. Vital organs were collected and bioluminescence (luciferase expression) and fluorescence (LNP distribution) were measured by an IVIS Spectrum imaging system. For cell uptake assay, the lung tissue fragments were treated with 1 mg/ml collagenase IV (Gibco) and 0.1 mg/ml DNase I (Sigma) for 30 min in RPMI 1640 medium. Samples were filtered through 70 μm filter and pre-treated with an anti-mouse CD16-CD32 antibody (BD, 553142) for 15 min. Cells were washed once and divided into two portions, one of which was stained with Fixable Viability Dye eF506 (eBioscience, 65-0866-18) FITC anti-CD45 (eBioscience, 11-0451-82) antibody. The other part was stained with PE-Cy7 anti-CD11c (Biolegend, 117318), eFluor450 anti-CD103 (eBioscience, 48-1031-82) PE-CF594 anti-Siglec-F (BD, 562757) and FITC anti-CD11b (Biolegend,101206) antibodies. AMs were gated as CD11c+ and Siglec-F+ cells. cDC1s were gated as CD11c + , Siglec-F- and CD103+ cells. CD11b+ cells were gated as CD11c + , Siglec-F-, CD103- and CD11b+ cells. Samples were analyzed on a flow cytometer (BD Fortessa or LSRII) and cellular uptake of LNP was evaluated using APC channel.

### In vivo toxicity evaluation

Balb/c mice were pre-treated with isoflurane and administrated with LNP via i.m., i.v. or i.n. at a dose of 2.5 μg RNA in 30 μl LNP per mouse. To monitor the systemic cytokine release, the serum was collected 6 h and 24 h after immunization. Mice received a second dose of LNP 7 days later and serum was collected as described above. The last dose of repeated administration was performed at day 14. Mice were sacrificed and whole blood was collected at the endpoint for blood routine examination (Servicebio). Analysis of cytokines was performed using a mouse inflammation CBA kit (BD, 552364) and IFN-α Elisa kit (Neobioscience, EMC035a). For the body weight evaluation, C57BL/6 J mice were intranasal immunized with LNP containing SIINFEKL-RFP circRNA (2.5 μg RNA per mouse) or left untreated and the body weight of each mouse was monitored at the indicated time point.

### Tumor model construction and immunization

For prophylactic tumor challenge studies, C57BL/6 J mice were immunized with LNP containing SIINFEKL-RFP circRNA (2.5 μg RNA per mouse) for two doses at an interval of 7 days. 30 or 60 days later, mice were challenged with 4 × 10^5^ B16-OVA-luciferase cells by intravenous injection. The tumor formation was monitored via IVIS Spectrum imaging system and the survival time of each mouse was recorded. For the therapeutic tumor model, C57BL/6 J mice received 5 × 10^5^ B16-OVA, 5 × 10^5^ B16-OVA-luciferase, 2 × 10^6^ B16-luciferase or 2.5 × 10^5^ LL/2 cells via intravenous injection. Mice were immunized with LNP containing SIINFEKL-RFP, B16 neoantigens, or LL/2 neoantigens circRNAs (2.5 μg circRNA per mouse) at the indicated time points. The tumor formation was monitored via IVIS Spectrum imaging system and the survival time at indicated experiments was recorded. The percentage of area occupied by lung metastases was calculated by Image J. The HE staining of tumor-bearing lung tissues was performed by Servicebio.

### Antigen-specific T-cell evaluation

To investigate the role of cDC1 on T cell priming, *WT* and *Batf3*^*−/−*^ mice were immunized with two doses of LNP containing SIINFEKL-RFP circRNA (2.5 μg RNA per mouse). In the experiment to inhibit lymphocyte egress from lymphoid tissues, FTY720 (MCE, 1 mg/kg) (or equal volume of normal saline) was intraperitoneal injected daily 1 day before RNA immunization. In the experiment to trace T cell proliferation, *WT*, *Batf3*^*−/−*^ or *CD11C-DTR* mice were pre-treated with cyclophosphamide (CTX, Sigma, 50 mg/kg) and diphtheria toxin (DT, Sigma, 4 μg/kg). Activated OT-1 cells were labeled with 5 μM CFSE (Selleck) for 15 min at 37 °C and washed with PBS two times. Labeled cells (1 × 10^6^/mouse) were transferred to mice via intravenous injection. After 6 h, mice were immunized with the SIINFEKL-RFP-coding circRNA vaccine. The lung tissues from vaccinated mice were collected at indicated time points as described in the figures. All the lung tissues from tumor-bearing or normal mice were digested into single cells and pre-treated with an anti-mouse Fc blocker. Samples were stained with Viability Dye eF506 (eBioscience, 65-0866-18), FITC anti-CD45 (eBioscience, 11-0451-82), PE anti-CD8 (Biolegend, 100708) and OVA Tetramer-SIINFEKL-APC (MBL, TS-5001-2C). For OT-1 transferred samples, cells were stained with AF780 anti-CD8 (eBioscience, 47-0081-80) and OVA Tetramer-SIINFEKL-PE (MBL, TS-5001-1C). All samples were analyzed on a flow cytometer (BD Fortessa or LSRII). To verify the origin of antigen-specific T cells, mice were intranasally administrated with two doses of vaccine at an interval of five days. At the endpoint, mice were intravenous injected with PE anti-CD3 (Biolegend, 100308) antibody. Three minutes later, mice were sacrificed, perfused with PBS, and lung tissues were quickly dissected, rinsed of free Ab, followed by digested into single cell. Cells were blocked with FC blocker and then stained with Fixable Viability Dye eFluor 780 (eBioscience, 65-0865-14), OVA Tetramer-SIINFEKL-APC (MBL, TS-5001-2C), FITC anti-CD8 (MBL, K0227-4). Samples were analyzed on a flow cytometer (BD).

### Adoptive cell transfer therapy

For combination therapy with adoptive T cell transfer and vaccine, *CD45.1, Rag1*^*−/ −*^ or *WT* mice were challenged with B16-OVA-luciferase, 5 × 10^5^ B16-OVA cells or 1 × 10^6^ EGFR-B16-luciferase cells, respectively. 1 × 10^6^ OT-1, 1 × 10^6^ OT-1-luciferase or 1 × 10^5^ CAR-T cells were intravenously injected into tumor-bearing *CD45.1, Rag1*^*−/−*^ or *WT* mice after CTX (50 mg/kg) pre-treatment. Intranasal immunization was performed at the indicated time points. In the experiment of *CD45.1* mice, the lung tissues were collected at the time point as described in the figures. For antigen-specific T cell detection, samples were stained with tetramer-SIINFEKL-PE (MBL, TS-5001-1C), FITC anti-CD8 (MBL, K0227-4), APC anti-CD45.1 (eBioscience, 17-0453-81), AF780 anti-CD45.2 (eBioscience, 47-0454-82). To investigate the memory phenotype of splenocytes from mice, 2 × 10^7^ splenocytes were transferred to *Rag1*^*−/−*^ mice, followed by 5 × 10^5^ B16-luciferase tumor rechallenge. The long-term survival was monitored.

### Statistical analysis

All the statistical analysis were performed by using GraphPad Prism 8. All data are presented as the mean ± SEM. The student’s t-test, one-way ANOVA, or two-way ANOVA with Tukey’s multiple comparisons test were performed for data analysis. For mouse survival curves, data were analyzed via Kaplan-Meier analysis. The level of significance was defined at *p < 0.05, **p < 0.01, ***p < 0.001, ****p < 0.0001.

## Supplementary information

Supplementary Materials

## Data Availability

The scRNA-seq data presented in the article have been uploaded to the GSA database with the identifier CRA022966. Other data are available in the main text or the supplementary materials.
