# Supplementary material for: Intranasal prime-boost RNA vaccination elicits potent T cell response for lung cancer therapy
Source: Signal Transduct Target Ther. 2025 Mar 24;10:101. doi: 10.1038/s41392-025-02191-1 (PMC11930932; doi:10.1038/s41392-025-02191-1)
Supplement: Supplementary file 1 — Supplementary Materials [file 41392_2025_2191_MOESM1_ESM.docx]

Supplementary Materials for

Intranasal prime-boost RNA vaccination elicits potent T cell response for lung cancer therapy

Hongjian Li,^1^ Yating Hu,^2^ Jingxuan Li,^1^ Jia He,^3^ Guocan Yu,^4^ Jiasheng Wang,^5^ Xin Lin,^1,5,6^*

Correspondence to: *linxin307@tsinghua.edu.cn*

**This PDF file includes:**

Materials and Methods

Figures. S1 to S13

Tables S1 to S2

Materials and Methods

Virus packaging

HEK293T cells were seeded onto 10 cm dishes till the confluency reached 70%. For each dish, 18 μg plasmid (12 μg gene-coding plasmid + 6 μg Pcl-Eco packaging plasmid) was added to 1 mL Opti-MEM medium, followed by the addition of 45 μL PEI (yeasen). After 15 min, the mixture was gently added to the dish. 12 hours later, the culture medium was removed and replenished with 10 mL pre-warmed DMEM medium (6% FBS+ penicillin/streptomycin). Virus-containing supernatants were collected at 48 hours and 72 hours after transfection. Samples were filtered through a 0.45 μm filter and precipitated via PEG-8000 at 4℃ for more than 12 hours, followed by centrifuging at 4000 rpm for 30 min. The supernatants were removed and the virus pellet was re-suspended in RIPM medium at 1/100 of the original volume and stored at -80℃.

Primary mouse T cell isolation and culture

For T cell activation, 24-well plates were pre-coated with 0.5 mL PBS containing anti-CD3 (BioXcell, BE0001-1) and anti-CD28 (BioXcell, BE0015-1) antibodies per well at 4℃ for more than 12 hours. Mice were sacrificed to collect the spleen tissue. Purified T cells were obtained by mouse T cell isolation kit (Stemcell, 19851) and seeded onto a pre-coated 24-well plate at a density of 2×10^6^/mL in 500 μL T cell medium (RIPM1640 medium+10%FBS+penicillin/streptomycin 1×NEAA+1×Sodium pyruvate+1×2-mercaptoethanol+50U/mL IL-2) per well for 24 hours. For virus infection, 5 μL virus with 0.5 μL polybrene (yeasen) were added to each well and cells were centrifuged at 1700 rpm for 1 hour. 12 hours later, cells were re-suspended and seeded onto 10 cm dishes at a density of 5×10^5^/mL in 10 mL T cell medium. Then, cells were cultured for another 24 hours before use. For T cells without the need for virus infection, cells were re-suspended in T cell medium at a density of 5×10^5^/mL and cultured for another 24 hours before use.

Preparation of CAR-T cells

OT-1 cells were isolated, activated and infected with anti-EGFR-CAR-coding virus as described above. 12 hours later, cells were re-suspended and seeded onto 10 cm dishes at a density of 5×10^5^/mL in 10 mL T cell medium for another 36 hours. The RFP positive cells were sorted via flow cytometry (BD Aria). Cell number were calculated and intravenously transferred back to the mice or co-cultured with target cells in vitro.

In vitro characterization of vaccine-responsive CAR-T cells

For coculture assay with CAR-T (effector) cells and EGFR-expressing B16 (target) cells, 2×10^4^ target cells were seeded at 96-well plate per well. 2×10^4^ OT-1 cells were added to the 96-well plate per well as the control group. 5×10^3^ or 2×10^4^ CAR-T cells were added to the plate per well to set as E: T (effector: target) = 1:4 or E: T = 1:1 group. Wells without T cells were set as blank group. Cells were cocultured for 24 hours and then washed with PBS for once. Luciferase activity was measured by a luciferase detection kit (Yeasen). Tumor cell lysis (%) = [1-(other groups / blank group)] × 100. For coculture assay with CAR-T cells and DCs, 5×10^4^ DC2.4 cells were seeded onto 48-well plate per well. Cells were transfected with different doses of RNA by using the Lipofectamine MessengerMax (Invitrogen). 24 hours later, cells were washed with PBS once and 5×10^4^ CAR-T cells were added to the wells to coculture for another 24 hours. Supernatant were collected for IFN-γ detection via mouse IFN-γ uncoated ELISA kit (Invitrogen). Cells were resuspended in PBS and stained with viability dye eF780 (eBioscience, 65-0865-14), FITC anti-CD69 (Biolegend, 104505) and BV421 anti-CD25 (Biolegend, 101923) antibodies. Samples were analyzed on a flow cytometer (BD).

RNA vaccine stability evaluation

SM102 based LNP formulations were employed to perform the stability assay. After completing the LNP preparation and dialysis, an equal volume of a 20% sucrose solution (w/w) was added, and the mixture was then filtered using a 0.22 μm filter. The formulations were initially stored at -20°C and then kept at 37°C or 4°C for various time points. At the endpoint, HEK293T cells were seeded onto 48-well plate and were transfected with LNPs encapsulated with D2GFP-coding circRNA or equal molar of its linear counterpart. 24 hours after transfection, cells were washed, stained with Fixable Viability Dye eFluor 780 (eBioscience, 65-0865-14) and analyzed on a flow cytometer (BD).

CD8 depletion assay

C57BL/6J mice received 5×10^5^ B16-OVA-luciferase cells via intravenous injection. Mice were immunized with LNP containing SIINFEKL-RFP circRNAs (2.5 μg circRNA per mouse) at day5 and day10. Anti-CD8 antibody (Selleck, A2102, 200 μg/mouse) was intraperitoneally injected every three days from day5. The tumor formation was monitored via IVIS Spectrum imaging system and mice were sacrificed at day14.

Immunofluorescence staining of antigen-specific T cells and APCs in vivo.

*Rag1^-/-^* mice received 5×10^5^ B16-OVA-luciferase cells via intravenous injection. At day6, activated 1×10^6^ OT-1 cells were intravenously transferred back to the mice. On the following day, mice were intranasally administrated with SIINFEKL-RFP circRNA vaccine. Mice were sacrificed and lung tissues were fixed in 4% paraformaldehyde and then sectioned for immunofluorescence staining via Servicebio.

T cell function analysis

CD45.1 mice were challenged with 5×10^5^ B16-OVA-luciferase. Activated 1×10^6^ OT-1 cells were intravenously injected into tumor-bearing mice after CTX (50 mg/kg) pre-treatment. Intranasal immunization was performed at the indicated time points. Lung tissues were collected at day 15 and single cell were obtained based on the methods above. 1×10^6^ cells were seeded onto 48-well plate in T cell medium with Brefeldin A (Biolegend, 420601). Cells were restimulated with SIINFEKL peptide (10 μg/mL) or left unstimulated for 4 hours. Cells were blocked with FC blocker and stained with Fixable Viability Dye eF506 (eBioscience, 65-0866-18), FITC anti-CD8 (MBL, K0227-4), AF780 anti-CD45.2 (eBioscience, 47-0454-82). Then samples were stained with PB anti-mouse IFN-γ (Biolegend, 505817) according to the protocol of Cytofix/Cytoperm Fixation/Permeabilization kit (BD, 554714). Samples were analyzed on a flow cytometer (BD).

Elispot assay

All the Elispot assay was performed following the protocols of the mouse IFN-γ pre-coated Elispot kit (Dakewe, 2210005). To monitor the induction of neoantigen-specific T cells, the RNA vaccine-encoded neoantigen peptides were synthesized by Genscript and the sequences of peptides were provided as follows: For LL/2 neoantigens, LSPRHYYSGYSSSL(Elfn2-P762L), LSPIHYSSA(Mastl-D366Y) and LTLHYRTL (Zscan21-H409L) were selected for T cell restimulation. For B16 antigens, SVYDFFVWL (Trp2), LAYLMKGL (Tnpo3-G504A) and QSLGFTYL (Atp11a-R552S) were selected for T cell restimulation. Single cell from lung tissues were isolated based on the digestion methods as mentioned above. 2×10^5^ cells were cultured in 96-well plate with mixed neoantigens (10 μg/ml) for 24 hours and then we monitored the formation of spots. For the experiment to evaluate the anti-tumor ability of OVA antigen loss tumor cells, B16-luciferase cells were pre-treated with 100IU IFN-γ (PeproTech) for 12 hours, followed by PBS wash two times. 2×10^4^ cells were co-cultured with 3×10^5^ splenocytes for another 36 hours and then we analyzed the spot formation. For the experiment to test the function of APCs for boosting T cells, lung tissues were collected 24 hours after being vaccinated with SIINFEKL-luciferase coding circRNA and digested into single cells as described above. Cells were pre-treated with an anti-mouse Fc blocker and stained as described above. Cells were sorted through BD FACS Aria by gating the markers of CD45- cells, AMs, CD103+ DCs and CD11b+ DCs. 5×10^3^ sorted cells were cocultured with 2×10^4^ OT-1 cells for 36 hours in pre-coated anti-IFN-γ Elispot plates. We monitored the formation of spots based on the protocol above. For the experiment to confirm the role of cDC1 present the OVA antigen at the lymph node, the mediastinal lymph nodes from vaccinated mice were collected 24 hours later. Tissues were digested using RPMI 1640 containing 0.2 mg/ml DNase I, 1.5 mg/ml collagenase IV and 10% heat-inactivated serum for 30 min at 37°C. Cells were blocked with FC blocker and stained with FITC anti-CD3 (Biolegend, 100306), FITC anti-CD19 (Biolegend, 101505), PE-Cy7 anti-CD11c (Biolegend, 117318), eFluor450 anti-CD103 (eBioscience, 48-1031-82). Cells were sorted based on FITC negative, PE-cy7 and eFluor450 double positive cells (BD Aria). 2000 sorted cells were cocultured with 1×10^4^ OT-1 cells for 24 hours in pre-coated anti-IFN-γ Elispot plates and monitored the formation of spots.

Single-cell RNA sequencing

The mouse model was performed as described above in the combination therapy assay. CD45.1 mice received with CD45.2 OT-1 and RFP-coding circRNA were defined as the control group (mock, n=3). CD45.1 mice received with CD45.2 OT-1 and SIINFEKL-RFP-coding circRNA were defined as the vaccine group (vac., n=3). All the lung tissues from the mice were digested into single cells and pre-treated with an anti-mouse Fc blocker. The samples from the same group were mixed for further staining. Cells were stained with Viability Dye eF506 (eBioscience, 65-0866-18), FITC anti-CD3 (Biolegend, 100306), APC anti-CD45.1 (eBioscience, 17-0453-81), AF780 anti-CD45.2 (eBioscience, 47-0454-82) and re-suspended in PBS buffer containing 10% FBS. Cells were sorted through BD FACS Aria by gating the CD3, CD45.1 positive and CD45.2 negative cells. BD Rhapsody system was used to capture the transcriptomic information of the single cells. Single-cell capture was achieved by random distribution of a single-cell suspension across >200,000 microwells through a limited dilution approach. Beads with oligonucleotide barcodes were added to saturation so that a bead was paired with a cell in a microwell. A cell-lysis buffer was added so that poly-adenylated RNA molecules could be hybridized with the beads. Beads were collected into a single tube for reverse transcription. Upon cDNA synthesis, each cDNA molecule was tagged on the 5′ end (that is, the 3′ end of a mRNA transcript) with a unique molecular identifier (UMI) and cell label indicating its cell of origin. Whole transcriptome libraries were prepared using the BD Rhapsody single-cell whole-transcriptome amplification workflow. In brief, the second strand cDNA was synthesized, followed by ligation of the WTA adaptor for universal amplification. Eighteen cycles of PCR were used to amplify the adaptor-ligated cDNA products. Sequencing libraries were prepared using random priming PCR of the whole-transcriptome amplification products to enrich the 3′ end of the transcripts linked with the cell label and UMI. Sequencing libraries were quantified using a High Sensitivity DNA chip (Agilent) on a Bioanalyzer 2200 and the Qubit High Sensitivity DNA assay (Thermo Fisher Scientific). All libraries were sequenced by DNBSEQ-T7 Sequencer (MGI) on a 150 bp paired-end run.

Single cell RNA data processing and analysis

scRNA-seq data analysis was performed by NovelBio Co., Ltd. with NovelBrain Cloud Analysis Platform. We applied fastp with default parameter filtering the adaptor sequence and removed the low-quality reads to achieve clean data. To quantify the gene expression of the single-cell data, we used STARsolo (version 2.7.10a) along with mouse genome mm10 (ensemble annotation version 100). The raw gene expression matrix was filtered, normalized, and clustered using R (version: 4.3.2). The quality control parameters were performed as follows: cells expressed less than 200 genes and 500 UMI counts with transcripts detected in less than 3 cells were removed. Besides, cells with a high mitochondrial genome transcript ratio (>0.2) were also filtered. Then the matrix was normalized and scaled by Seurat’s SCTransform function (version: 0.4.1). Fast integration using reciprocal PCA (RPCA) was used to integrate cells between two samples. After integration, dimensionality reduction and unsupervised clustering were performed following Seurat’s tutorial (version: 4.3.0). Briefly, PCA was applied first and the top 30 dimensions were loaded for UMAP dimensionality reduction. To find clusters, the same PCs were imported into Seurat’s FindAllClusters function with a resolution of 0.6 and the cell types were manually annotated based on the prior knowledge of marker genes.

The differential gene expression and Gene Ontology (GO) enrichment analysis

The differential gene expression analysis between two samples in activated CD4 and effector CD8 clusters were identified by Seurat’s FindMarkers function, respectively. Genes with adjusted p value less than 0.05 (p_val_adj) and average log Fold Change (avg_logFC) larger than 1 were considered as the differentially expressed genes (DEGs). GO enrichment analysis was performed on DEGs using clusterProfiler’s enrichGO function (version: 4.10.0).

Calculation of the signature score

To evaluate the cytotoxic function between two samples in per clusters, the cytotoxicity gene set was defined as a family of Granzymes (Gzma, Gzmb, Gzmc, Gzmd, Gzme, Gzmf, Gzmg, Gzmk, Gzmm) in this study. The signature enrichment score of the cytotoxicity gene set was calculated by AUCell’s AUCell_run function (version: 1.24.0).


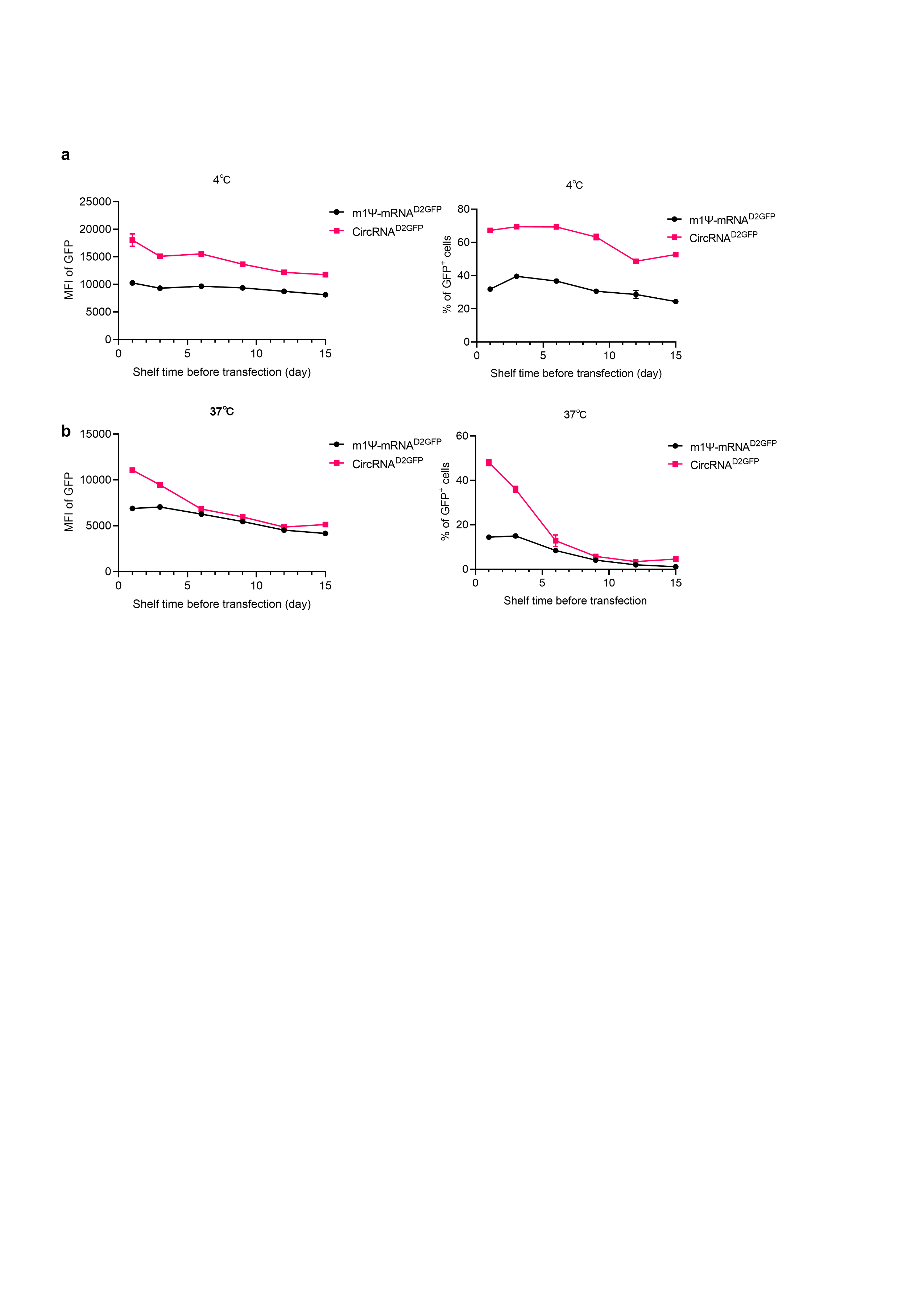


**Figure. S1.**

Statistical results of GFP positive cells and the mean fluorescence intensity (MFI) of HEK293T cells after transfected with D2GFP-coding circRNA and its linear counterpart stored at 4°C (**a**) or 37°C (**b**) over different periods. Data are represented as mean ± SEM.


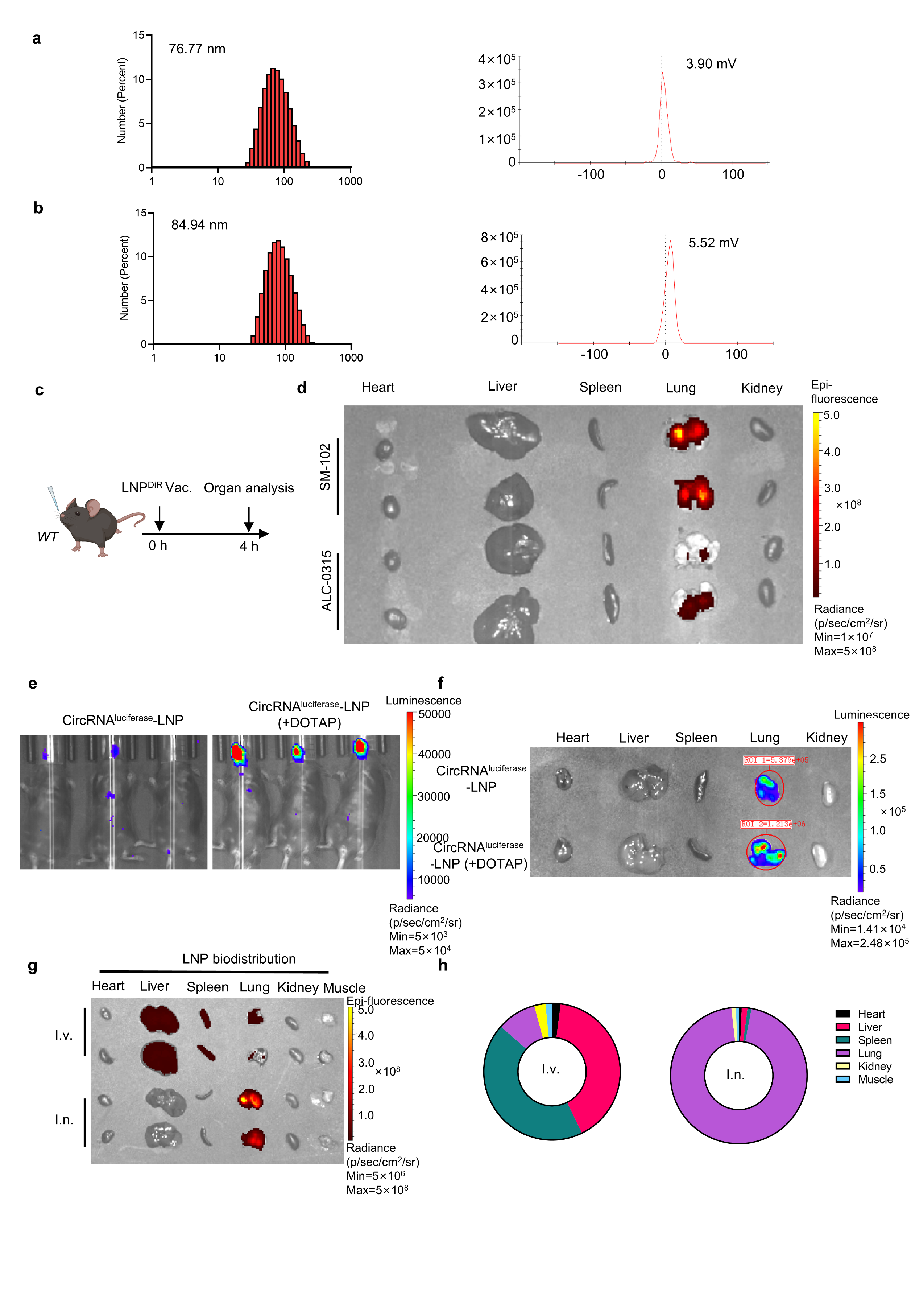


**Figure. S2.**

LNP components with DOTAP and SM102 initiates protein expression at the lung tissue and nasal site. **a, b** Size distributions and zeta potential of the SM102-LNP^DiR^ (a) and ALC-0315 LNP^DiR^ (b) vaccine. **c** Timeline of the experiment designed to evaluate the lung accumulation of different LNP complexes. LNPs were intranasally administrated into mice followed by organ analysis. **d** Imaging results based on the fluorescence intensity of DiR at different tissues. DiR was mixed with LNPs containing different clinical-approved ionizable lipids. **e** Imaging results of luciferase protein expression 4 hours after administrated with circRNA-LNP complex with or without DOTAP. **f** Luciferase expression among various organs of the representative mice in e. CircRNA was encapsulated with DOTAP-containing LNP and administrated to mice followed by organ analysis. Imaging results (**g**) and statistical analysis (**h**) of DOTAP-containing LNP biodistribution among various organs (related to Fig. 1e-g). I.v., intravenous injection group. I.n., intranasal administration group.


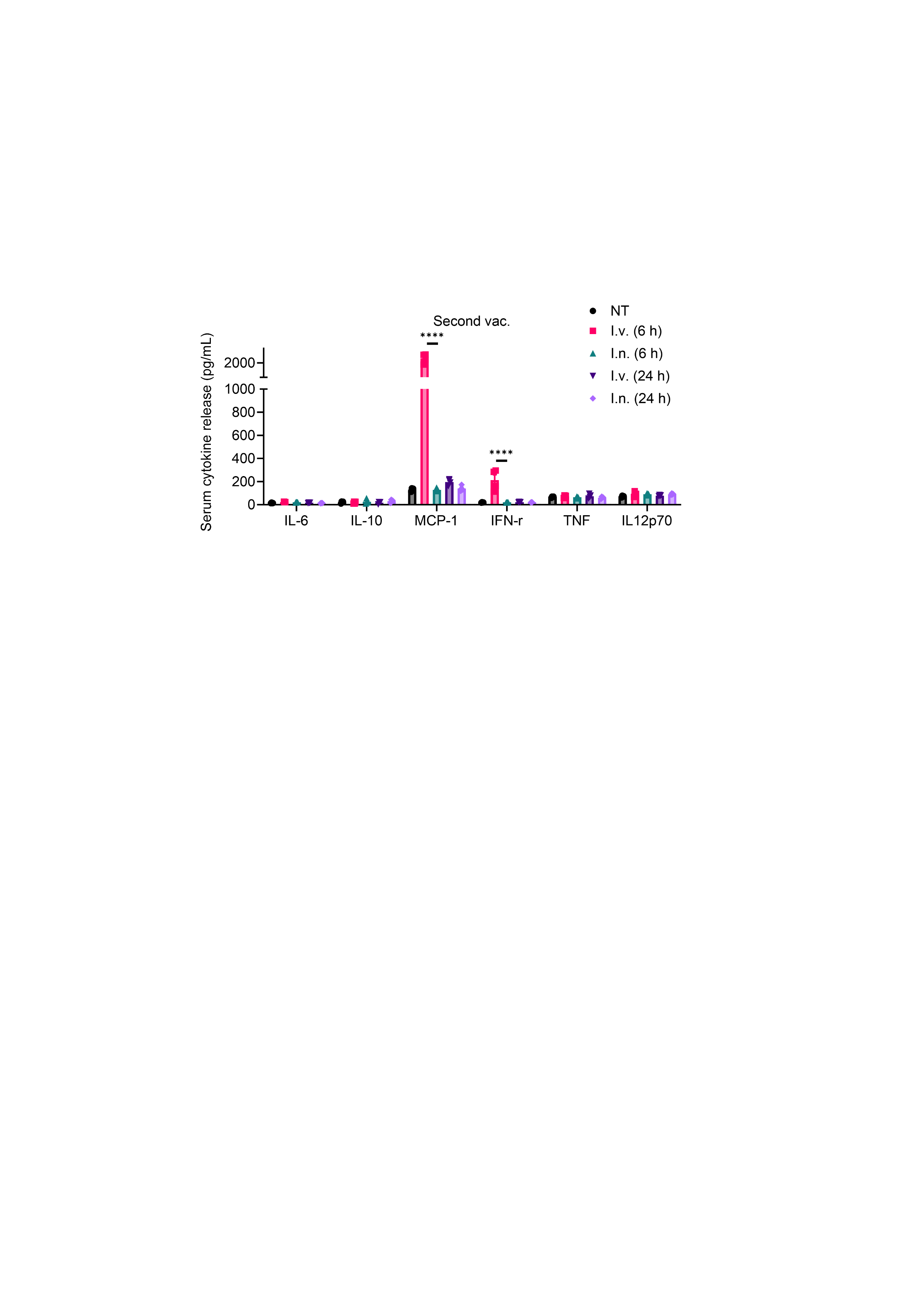


**Figure. S3.**

Serum cytokine release after second immunization with circRNA vaccine tested by mouse inflammation kit.


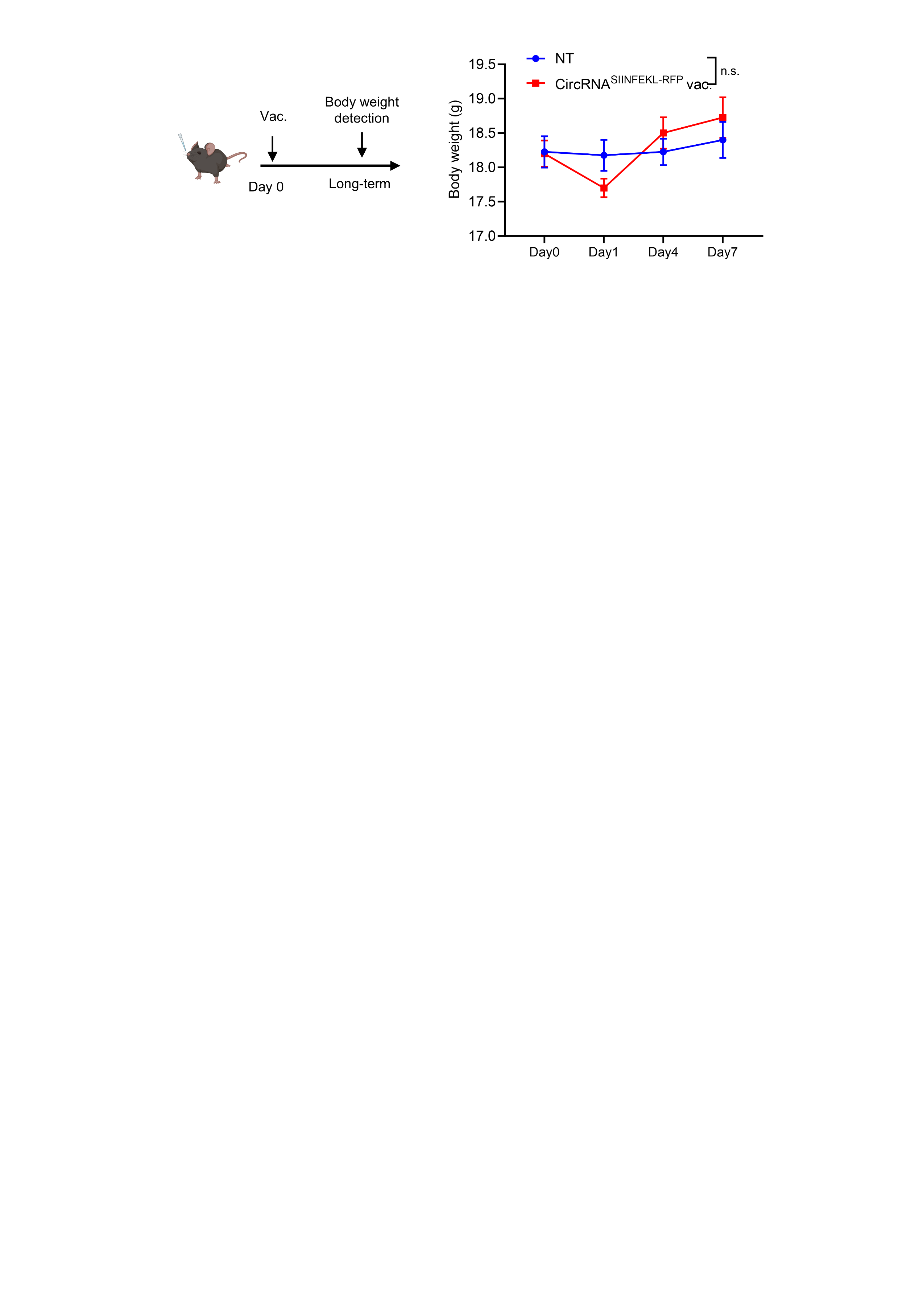


**Figure. S4.**

Statistical results of the body weight change after intranasal vaccination (n = 4 for each group). Data are represented as mean ± SEM.


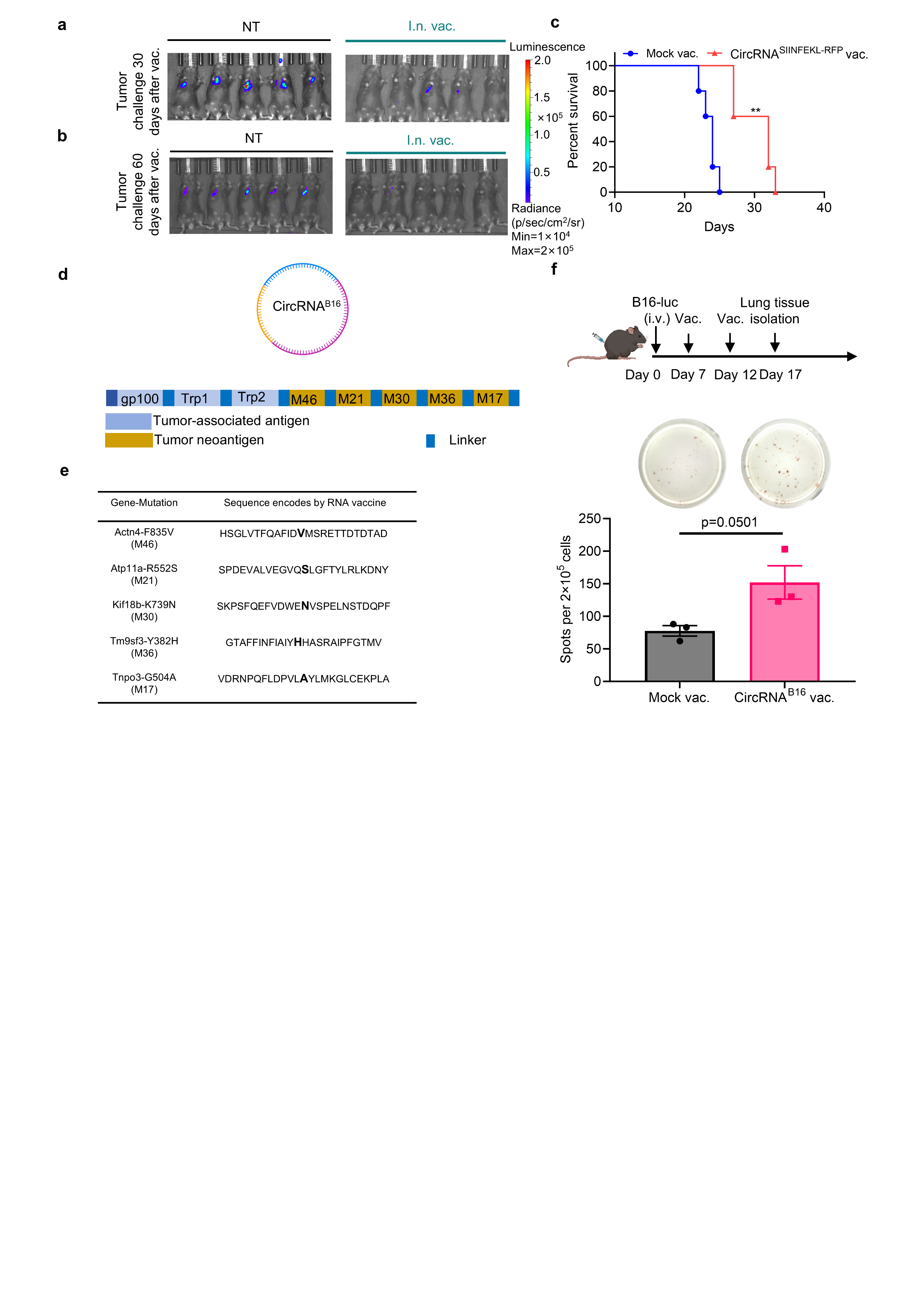


**Figure. S5.**

Intranasal circRNA inhibits tumor growth in B16-OVA and B16 lung cancer model. **a, b** Bioluminescence images of the experiments to evaluate the protective immunity induced by circRNA vaccine. Images were taken 6 days after tumor rechallenge. **c** Survival curves of the experiments. Mice were immunized with SIINFEKL-coding circRNA twice at an interval of 7 days, followed by rechallenging B16-OVA cells 30 days after the second vaccination. **d** Scheme of the B16 neoantigen-coding circRNA. **e** List of B16 neoantigens for the RNA vaccine design. **f** Images and statistical results of IFN-γ Elispot assay after culture cells from lung tissues loaded with B16 peptide mixture. Data were analyzed by Student’s t test and represented as mean ± SEM.


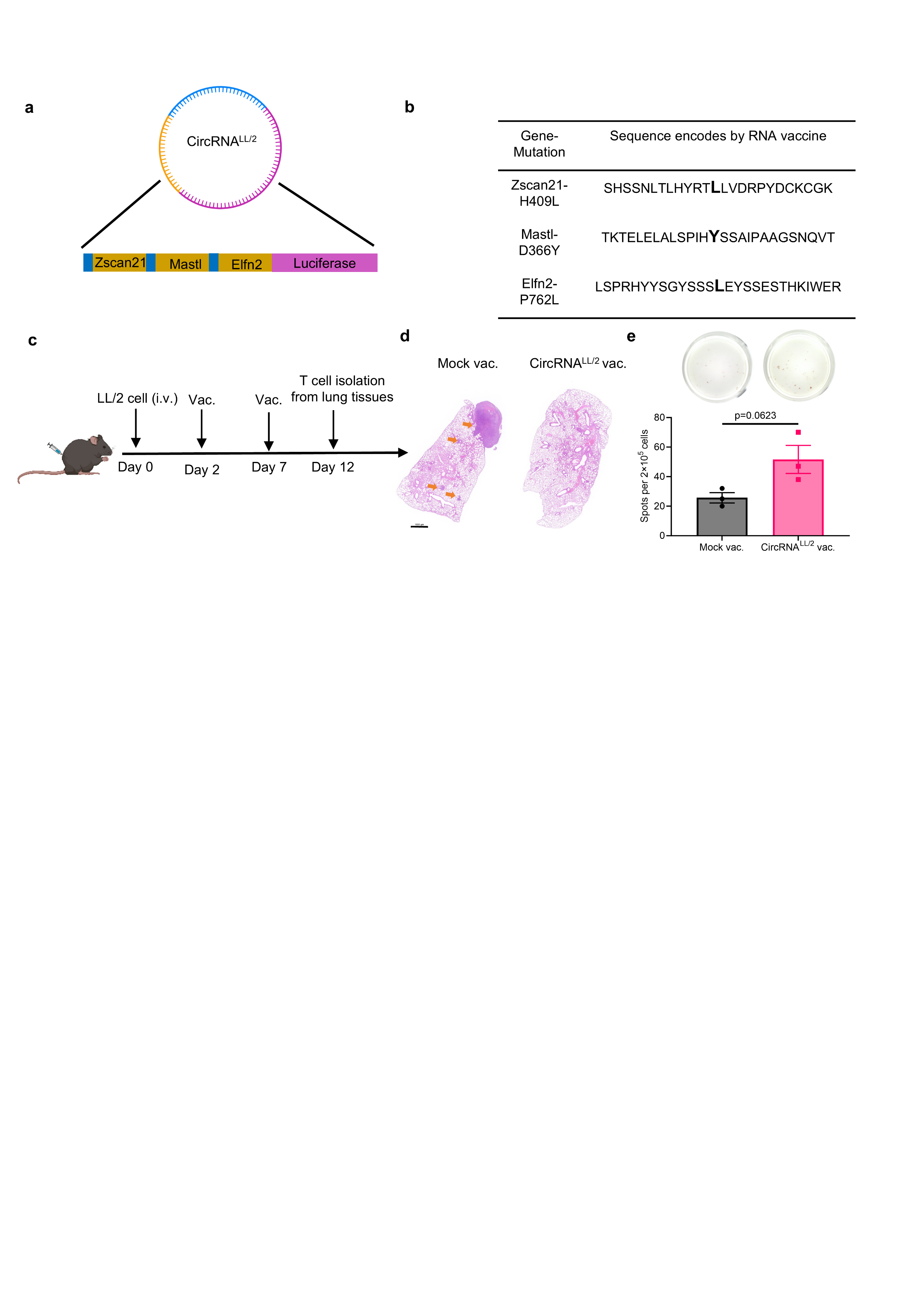


**Figure. S6.**

Intranasal circRNA inhibits tumor growth and induces tumor-specific T cell response in LL/2 lung cancer model. **a** Scheme of the LL/2 neoantigen-coding circRNA. **b** List of LL/2 neoantigens for the RNA vaccine design. **c** Timeline of the experiments to test the anti-tumor ability of intranasal circRNA vaccine in LL/2 lung cancer model. Mice were challenged with LL/2 cells, followed by LL/2 neoantigen-coding circRNA immunization. Mice (n=3) were sacrificed on day 12 to evaluate the tumor growth and T cell response to tumor cells. **d** Representative images of HE staining to evaluate the tumor growth at day 12. Scare bar, 1000 μm. **e** Images and statistical results of IFN-γ Elispot assay after culture cells from lung tissues loaded with LL/2 peptide mixture. Data were analyzed by Student’s t test and represented as mean ± SEM.


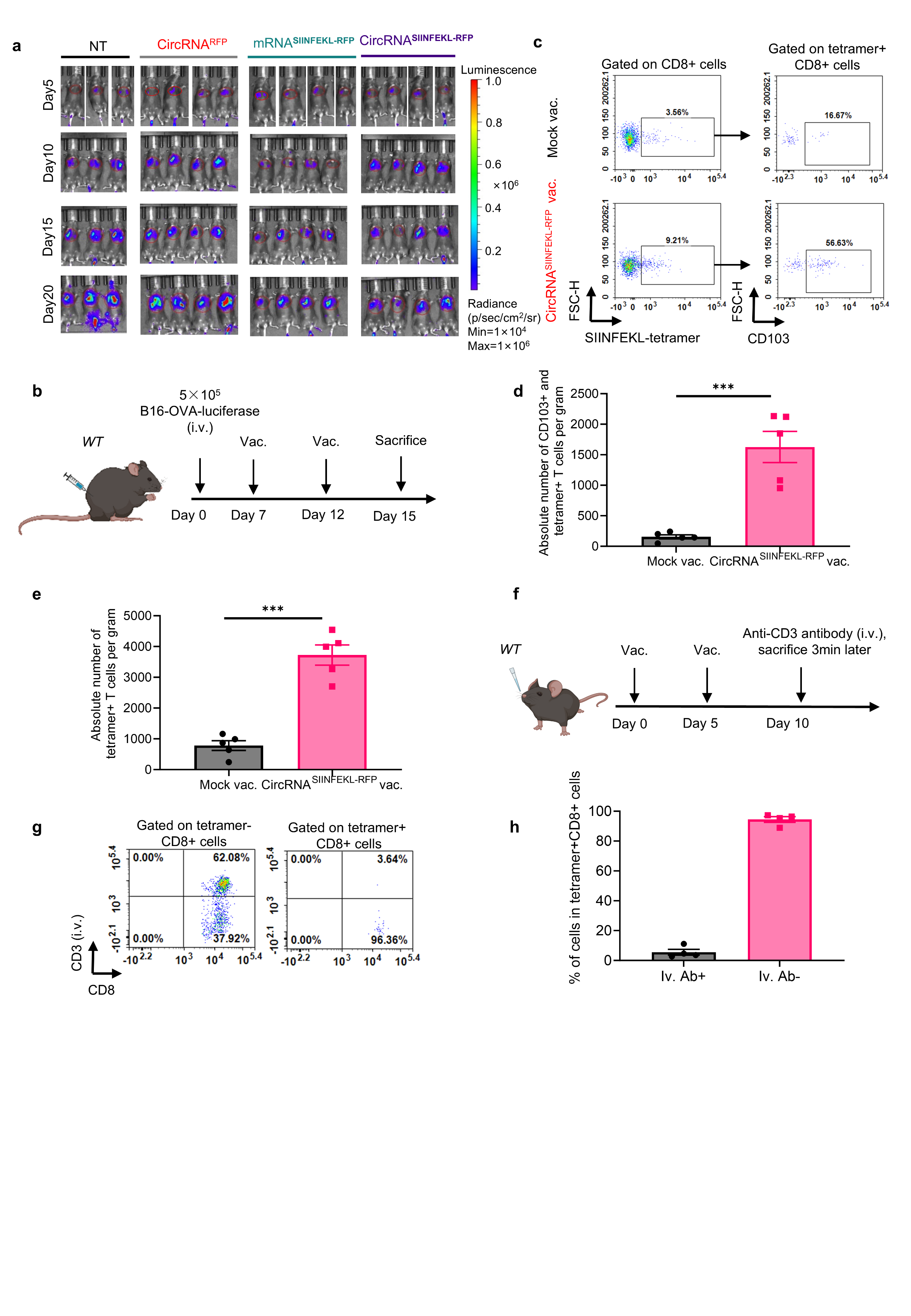


**Figure. S7.**

**a** Bioluminescence images of the mice with different treatments of the experiments in Fig. 3a. **b** Timeline of the experiment to analyze antigen-specific T cells in tumor-bearing mice. **c-e** Representative plots and statistical results of the tetramer positive CD8 T cells and tetramer/CD103 double positive CD8 T cells. **f** Timeline of the experiment to analyze antigen-specific T cells based on in-vivo staining method. **g, h** Representative plots (g) and statistical results (h) of the ratio of i.v. antibody positive cells within antigen-specific CD8 T cells. All data were analyzed by Student’s t test and represented as mean ± SEM.


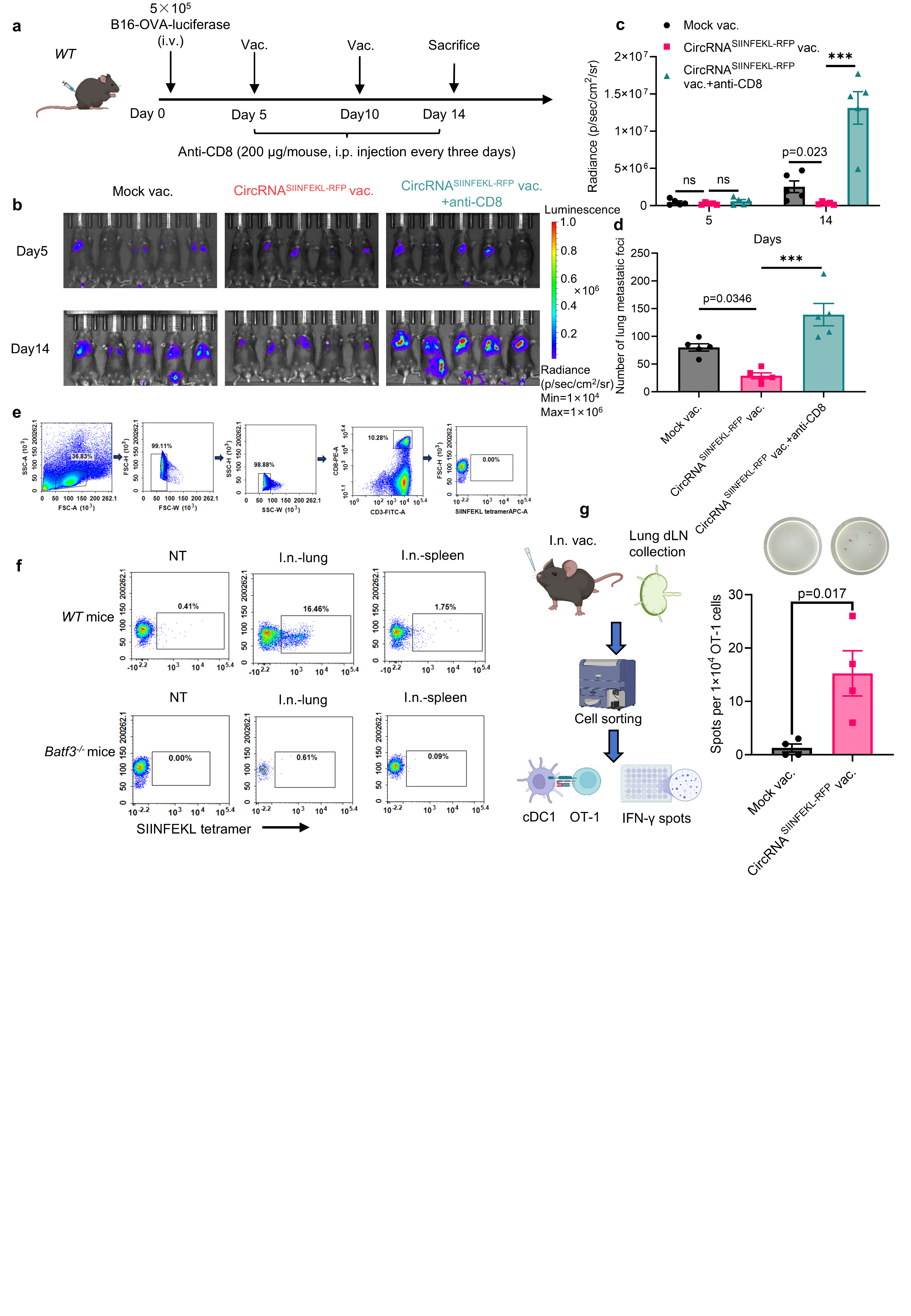


**Figure. S8.**

**a** Timeline of the experiment to evaluate the role of CD8 T cell in tumor control after vaccination. **b, c** Bioluminescence images (b) and statistical results (c) of average radiance at different time points. **d** Statistical results of the lung metastatic foci numbers in each group. Data were analyzed by one-way ANOVA with Tukey’s multiple comparisons test. **e,f** Gating strategy (e) and representative plots (f) of the experiments in Fig. 3g. **g** Images and statistical results of IFN-γ Elispot assay after culture with cDC1s from the mediastinal lymph node and antigen-specific OT-1 cells. Data were analyzed by Student’s t test. All data were represented as mean ± SEM.

**
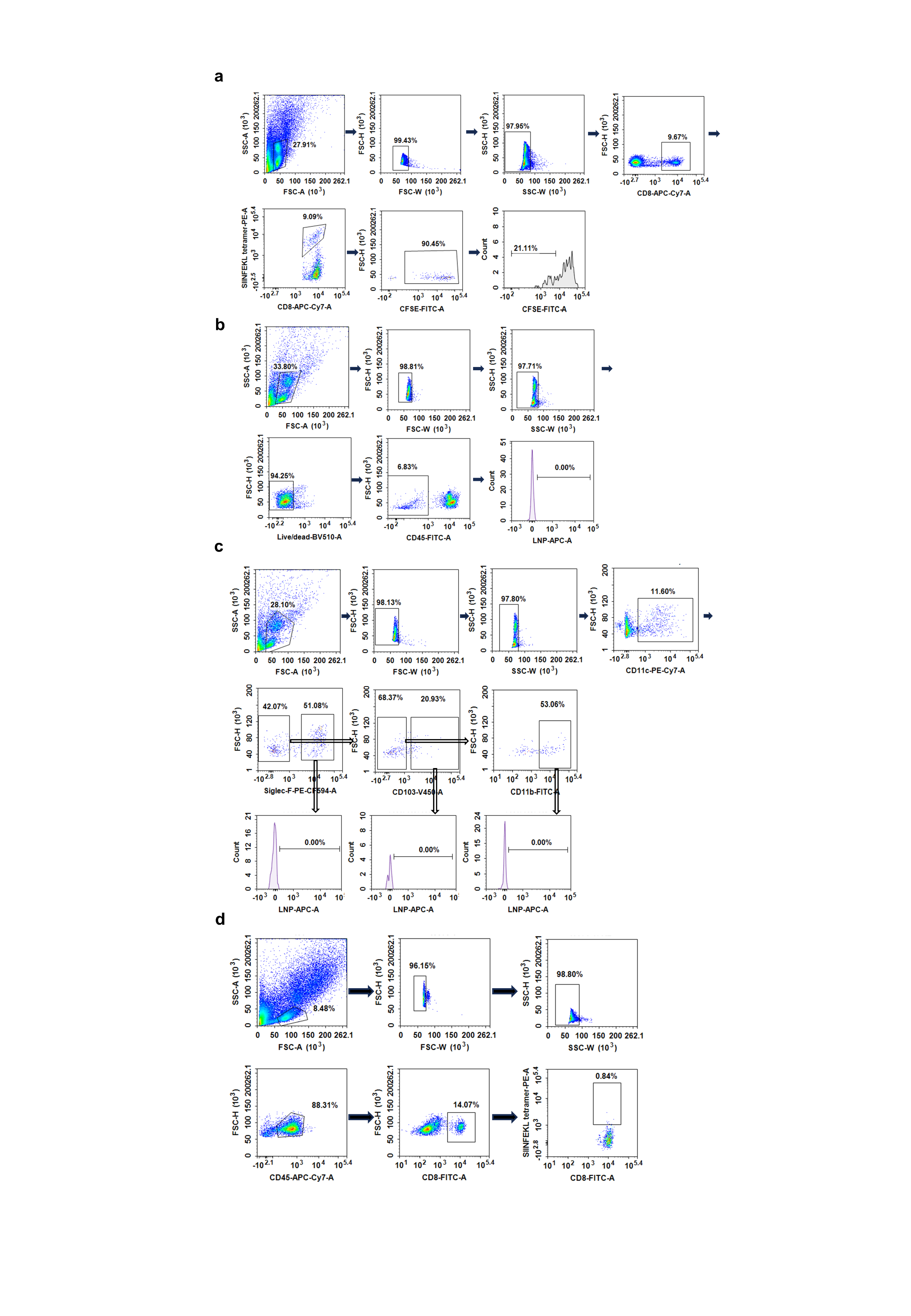
**

**Figure. S9.**

Gating strategy of the experiments in Fig. 4b (**a**), Fig. 4d (**b, c**) and Fig. 4j (**d**).


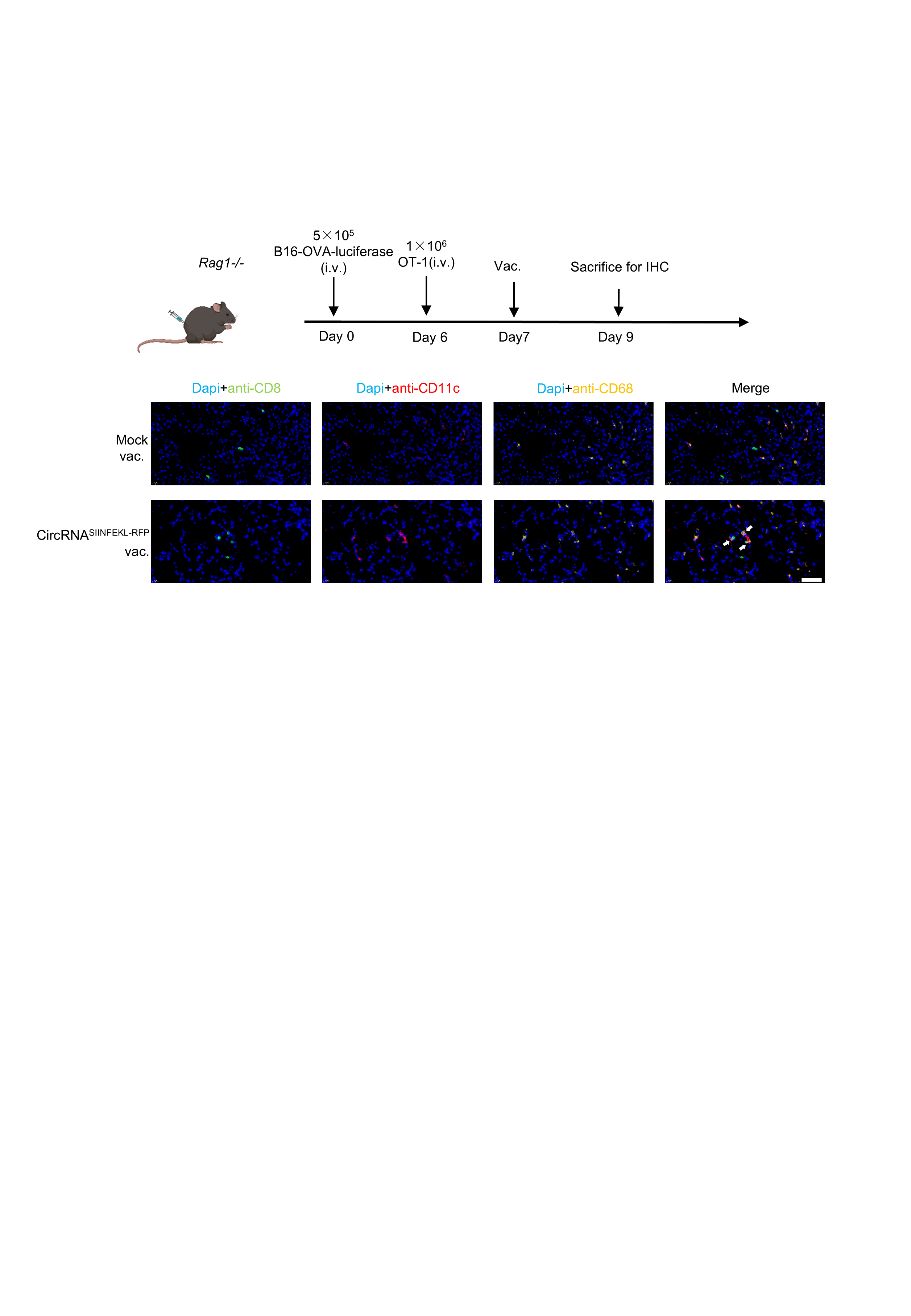


**Figure. S10.**

Representative IF staining images of lung tissues from tumor-bearing *Rag1^-/-^* mice after antigen-specific T cell transfer and vaccination. Scare bar, 60 μm.

**
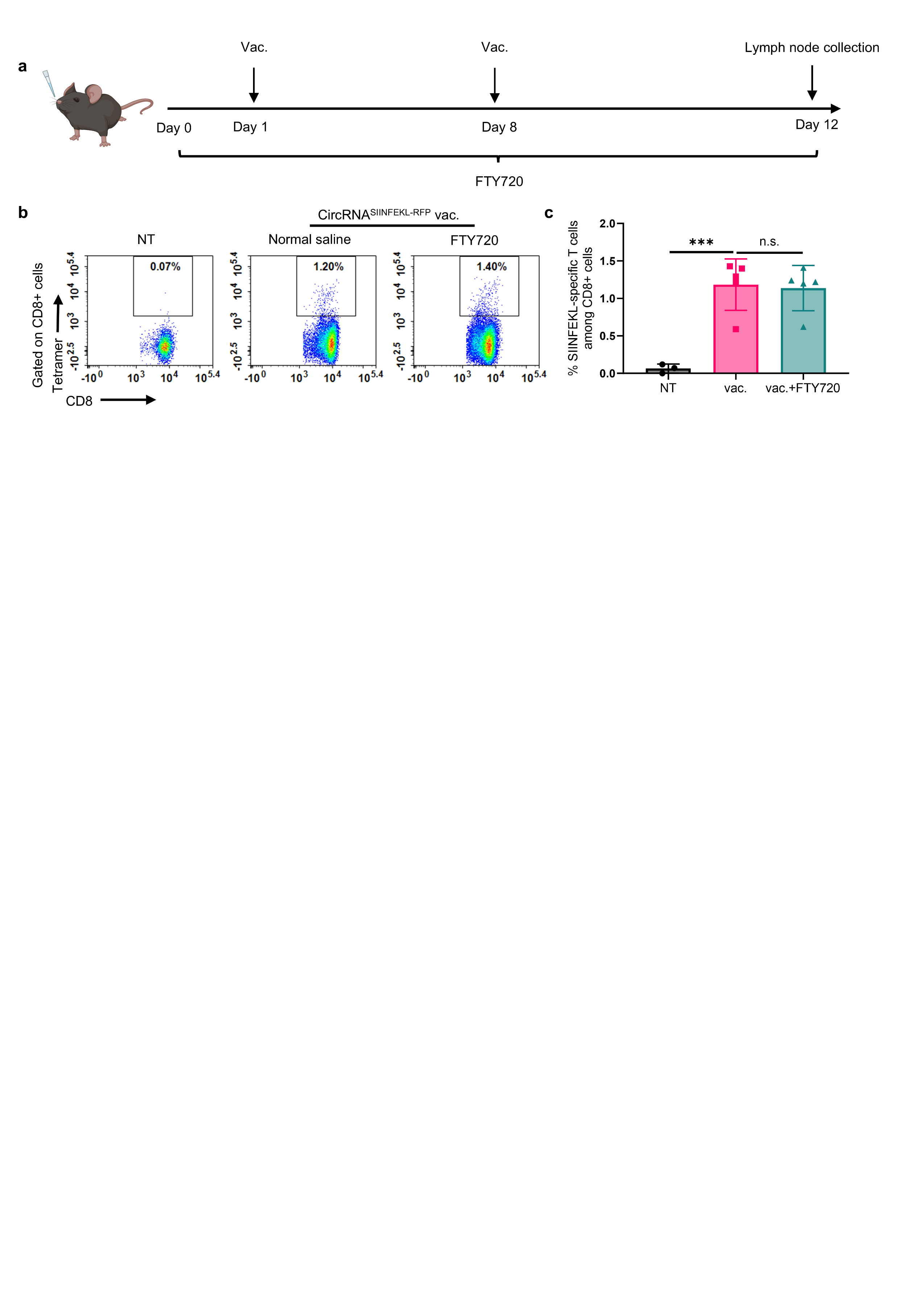
**

**Figure. S11.**

Intranasal circRNA vaccine can prime antigen-specific T cells at lymph node. **a** Timeline of the experiment to explore whether vaccine can prime T cells at the mediastinal lymph node. Mice were treated with FTY720 during the prime-boost process or normal saline as control group. The ratio of antigen-specific T cells was analyzed. **b, c** Representative plots (**b**) and statistical results (**c**) of the antigen-specific T cells with different treatments.


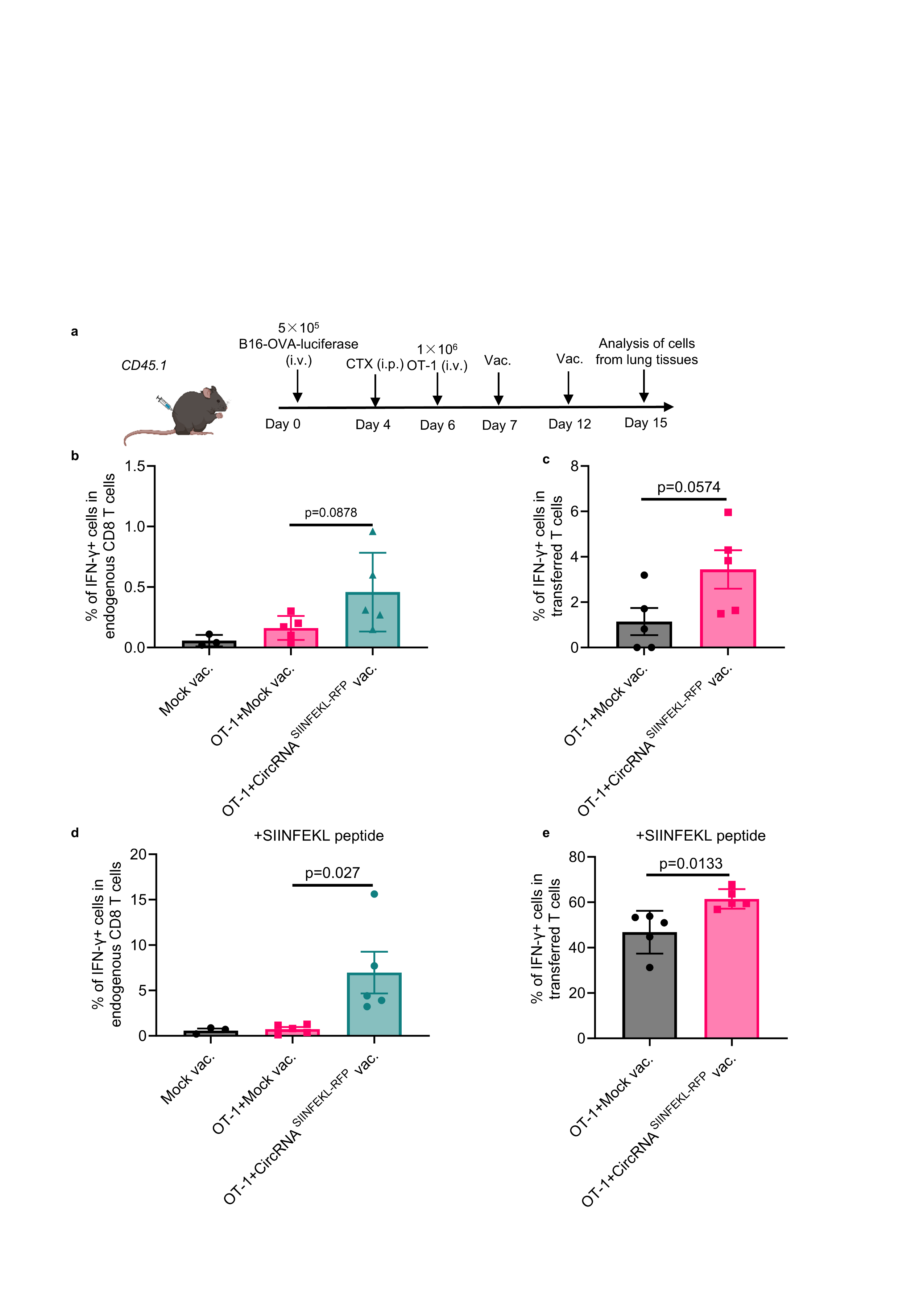


**Figure. S12.**

Detection of endogenous and transferred T cell function after intranasal vaccination. **a** Timeline of the experiment. **b, c** Statistical results of IFN-γ+ endogenous (b) or transferred (c) CD8 T cells. **d, e** Statistical results of IFN-γ+ endogenous (d) or transferred (e) CD8 T cells after in-vitro restimulation with SIINFEKL peptide. All data were analyzed by Student’s t test and represented as mean ± SEM.


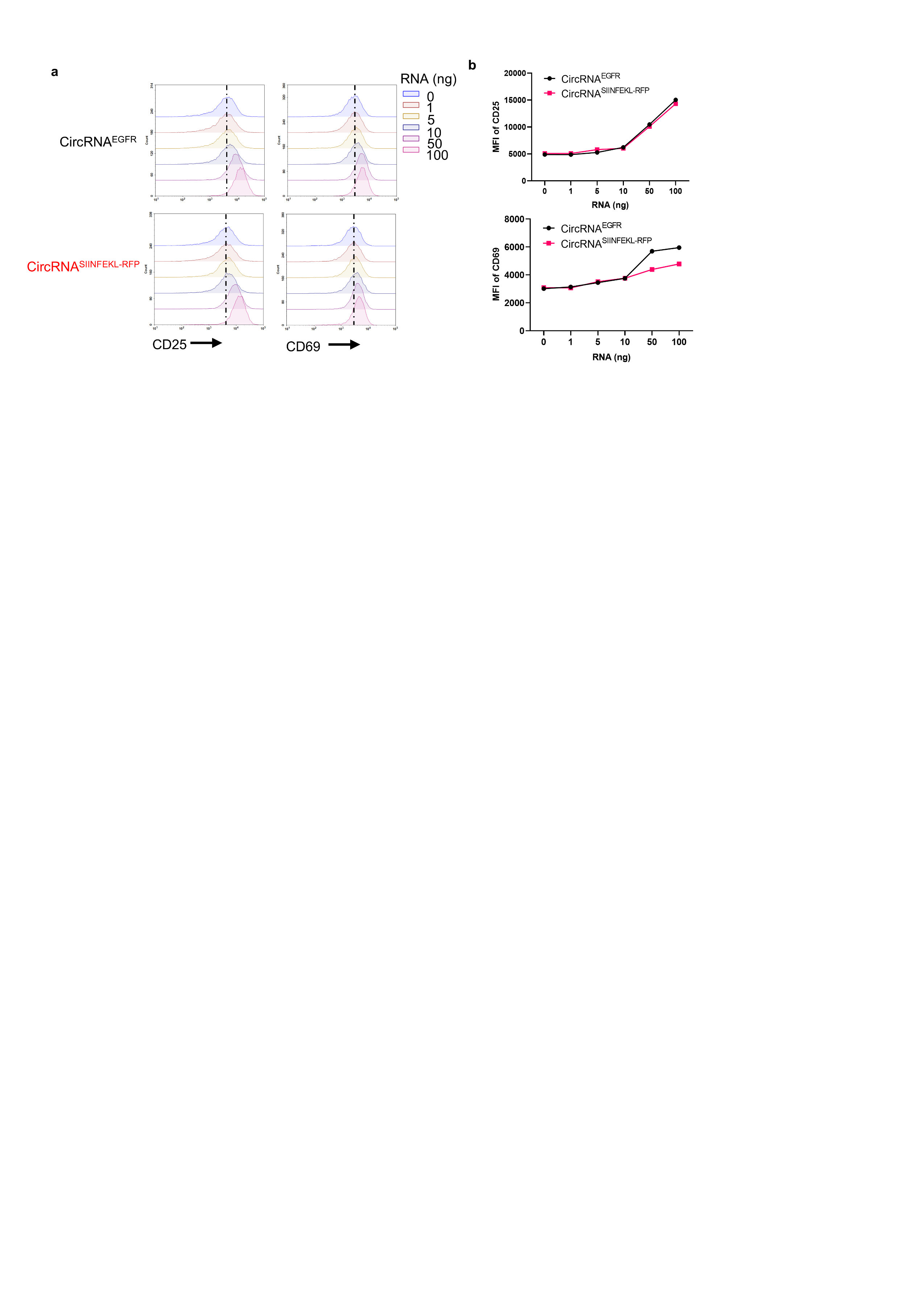


**Figure. S13.**

Detection of the vaccine-responsive CAR-T cell activation after coculture with DC2.4 cells. **a** Representative flow cytometry histogram of CD25 and CD69 expression on T cells. **b** Statistical results of median fluorescence intensity (MFI).

**Table S1.**

Nucleotide sequences of the plasmids used in the study.

| Region name | Template sequence |
| --- | --- |
| T7 promoter | taatacgactcactatagg |
| Arm and intron 1 | gggagaccctcgaccgtcgattgtccactggtcaacaatagatgacttacaactaatcggaaggtgcagagactcgacgggagctaccctaacgtcaagacgagggtaaagagagagtccaattctcaaagccaataggcagtagcgaaagctgcaagagaatg |
| Exon and spacer 1 | aaaatccgttgaccttaaacggtcgtgtgggttcaagtccctccacccccacgccggaaacgcaatagccgaaaaacaaaaaacaaaaaaaacaaaaaaaaaaccaaaaaaacaaaacaca |
| CVB3 IRES | TTAAAACAGCCTGTGGGTTGATCCCACCCACAGGCCCATTGGGCGCTAGCACTCTGGTATCACGGTACCTTTGTGCGCCTGTTTTATACCCCCTCCCCCAACTGTAACTTAGAAGTAACACACACCGATCAACAGTCAGCGTGGCACACCAGCCACGTTTTGATCAAGCACTTCTGTTACCCCGGACTGAGTATCAATAGACTGCTCACGCGGTTGAAGGAGAAAGCGTTCGTTATCCGGCCAACTACTTCGAAAAACCTAGTAACACCGTGGAAGTTGCAGAGTGTTTCGCTCAGCACTACCCCAGTGTAGATCAGGTCGATGAGTCACCGCATTCCCCACGGGCGACCGTGGCGGTGGCTGCGTTGGCGGCCTGCCCATGGGGAAACCCATGGGACGCTCTAATACAGACATGGTGCGAAGAGTCTATTGAGCTAGTTGGTAGTCCTCCGGCCCCTGAATGCGGCTAATCCTAACTGCGGAGCACACACCCTCAAGCCAGAGGGCAGTGTGTCGTAACGGGCAACTCTGCAGCGGAACCGACTACTTTGGGTGTCCGTGTTTCATTTTATTCCTATACTGGCTGCTTATGGTGACAATTGAGAGATCGTTACCATATAGCTATTGGATTGGCCATCCGGTGACTAATAGAGCTATTATATATCCCTTTGTTGGGTTTATACCACTTAGCTTGAAAGAGGTTAAAACATTACAATTCATTGTTAAGTTGAATACAGCAAA |
| Exon and spacer 2 | aaaaaacaaaaaacaaaacggctattatgcgttaccggcgagacgctacggactta |
| Arm and intron 2 | aataattgagccttaaagaagaaattctttaagtggatgctctcaaactcagggaaacctaaatctagttatagacaaggcaatcctgagccaagccgaagtagtaattagtaagaccagtggacaatcgacggataacagcatatctag |
| ORF of SIINFEKL-luciferase | ATGCTTGAGCAGCTTGAGAGTATAATCAACTTTGAAAAACTGACTGAATGGACCAGTggGGATCCACCGGTCATGGAAGACGCCAAAAACATAAAGAAAGGCCCGGCGCCATTCTATCCGCTGGAAGATGGAACCGCTGGAGAGCAACTGCATAAGGCTATGAAGAGATACGCCCTGGTTCCTGGAACAATTGCTTTTACAGATGCACATATCGAGGTGGACATCACTTACGCTGAGTACTTCGAAATGTCCGTTCGGTTGGCAGAAGCTATGAAACGATATGGGCTGAATACAAATCACAGAATCGTCGTATGCAGTGAAAACTCTCTTCAATTCTTTATGCCGGTGTTGGGCGCGTTATTTATCGGAGTTGCAGTTGCGCCCGCGAACGACATTTATAATGAACGTGAATTGCTCAACAGTATGGGCATTTCGCAGCCTACCGTGGTGTTCGTTTCCAAAAAGGGGTTGCAAAAAATTTTGAACGTGCAAAAAAAGCTCCCAATCATCCAAAAAATTATTATCATGGATTCTAAAACGGATTACCAGGGATTTCAGTCGATGTACACGTTCGTCACATCTCATCTACCTCCCGGTTTTAATGAATACGATTTTGTGCCAGAGTCCTTCGATAGGGACAAGACAATTGCACTGATCATGAACTCCTCTGGATCTACTGGTCTGCCTAAAGGTGTCGCTCTGCCTCATAGAACTGCCTGCGTGAGATTCTCGCATGCCAGAGATCCTATTTTTGGCAATCAAATCATTCCGGATACTGCGATTTTAAGTGTTGTTCCATTCCATCACGGTTTTGGAATGTTTACTACACTCGGATATTTGATATGTGGATTTCGAGTCGTCTTAATGTATAGATTTGAAGAAGAGCTGTTTCTGAGGAGCCTTCAGGATTACAAGATTCAAAGTGCGCTGCTGGTGCCAACCCTATTCTCCTTCTTCGCCAAAAGCACTCTGATTGACAAATACGATTTATCTAATTTACACGAAATTGCTTCTGGTGGCGCTCCCCTCTCTAAGGAAGTCGGGGAAGCGGTTGCCAAGAGGTTCCATCTGCCAGGTATCAGGCAAGGATATGGGCTCACTGAGACTACATCAGCTATTCTGATTACACCCGAGGGGGATGATAAACCGGGCGCGGTCGGTAAAGTTGTTCCATTTTTTGAAGCGAAGGTTGTGGATCTGGATACCGGGAAAACGCTGGGCGTTAATCAAAGAGGCGAACTGTGTGTGAGAGGTCCTATGATTATGTCCGGTTATGTAAACAATCCGGAAGCGACCAACGCCTTGATTGACAAGGATGGATGGCTACATTCTGGAGACATAGCTTACTGGGACGAAGACGAACACTTCTTCATCGTTGACCGCCTGAAGTCTCTGATTAAGTACAAAGGCTATCAGGTGGCTCCCGCTGAATTGGAATCCATCTTGCTCCAACACCCCAACATCTTCGACGCAGGTGTCGCAGGTCTTCCCGACGATGACGCCGGTGAACTTCCCGCCGCCGTTGTTGTTTTGGAGCACGGAAAGACGATGACGGAAAAAGAGATCGTGGATTACGTCGCCAGTCAAGTAACAACCGCGAAAAAGTTGCGCGGAGGAGTTGTGTTTGTGGACGAAGTACCGAAAGGTCTTACCGGAAAACTCGACGCAAGAAAAATCAGAGAGATCCTCATAAAGGCCAAGAAGGGCGGAAAGATCGCCGTGTGA |
| ORF of luciferase | ATGGAAGACGCCAAAAACATAAAGAAAGGCCCGGCGCCATTCTATCCGCTGGAAGATGGAACCGCTGGAGAGCAACTGCATAAGGCTATGAAGAGATACGCCCTGGTTCCTGGAACAATTGCTTTTACAGATGCACATATCGAGGTGGACATCACTTACGCTGAGTACTTCGAAATGTCCGTTCGGTTGGCAGAAGCTATGAAACGATATGGGCTGAATACAAATCACAGAATCGTCGTATGCAGTGAAAACTCTCTTCAATTCTTTATGCCGGTGTTGGGCGCGTTATTTATCGGAGTTGCAGTTGCGCCCGCGAACGACATTTATAATGAACGTGAATTGCTCAACAGTATGGGCATTTCGCAGCCTACCGTGGTGTTCGTTTCCAAAAAGGGGTTGCAAAAAATTTTGAACGTGCAAAAAAAGCTCCCAATCATCCAAAAAATTATTATCATGGATTCTAAAACGGATTACCAGGGATTTCAGTCGATGTACACGTTCGTCACATCTCATCTACCTCCCGGTTTTAATGAATACGATTTTGTGCCAGAGTCCTTCGATAGGGACAAGACAATTGCACTGATCATGAACTCCTCTGGATCTACTGGTCTGCCTAAAGGTGTCGCTCTGCCTCATAGAACTGCCTGCGTGAGATTCTCGCATGCCAGAGATCCTATTTTTGGCAATCAAATCATTCCGGATACTGCGATTTTAAGTGTTGTTCCATTCCATCACGGTTTTGGAATGTTTACTACACTCGGATATTTGATATGTGGATTTCGAGTCGTCTTAATGTATAGATTTGAAGAAGAGCTGTTTCTGAGGAGCCTTCAGGATTACAAGATTCAAAGTGCGCTGCTGGTGCCAACCCTATTCTCCTTCTTCGCCAAAAGCACTCTGATTGACAAATACGATTTATCTAATTTACACGAAATTGCTTCTGGTGGCGCTCCCCTCTCTAAGGAAGTCGGGGAAGCGGTTGCCAAGAGGTTCCATCTGCCAGGTATCAGGCAAGGATATGGGCTCACTGAGACTACATCAGCTATTCTGATTACACCCGAGGGGGATGATAAACCGGGCGCGGTCGGTAAAGTTGTTCCATTTTTTGAAGCGAAGGTTGTGGATCTGGATACCGGGAAAACGCTGGGCGTTAATCAAAGAGGCGAACTGTGTGTGAGAGGTCCTATGATTATGTCCGGTTATGTAAACAATCCGGAAGCGACCAACGCCTTGATTGACAAGGATGGATGGCTACATTCTGGAGACATAGCTTACTGGGACGAAGACGAACACTTCTTCATCGTTGACCGCCTGAAGTCTCTGATTAAGTACAAAGGCTATCAGGTGGCTCCCGCTGAATTGGAATCCATCTTGCTCCAACACCCCAACATCTTCGACGCAGGTGTCGCAGGTCTTCCCGACGATGACGCCGGTGAACTTCCCGCCGCCGTTGTTGTTTTGGAGCACGGAAAGACGATGACGGAAAAAGAGATCGTGGATTACGTCGCCAGTCAAGTAACAACCGCGAAAAAGTTGCGCGGAGGAGTTGTGTTTGTGGACGAAGTACCGAAAGGTCTTACCGGAAAACTCGACGCAAGAAAAATCAGAGAGATCCTCATAAAGGCCAAGAAGGGCGGAAAGATCGCCGTGTGA |
| ORF of SIINFEKL-RFP | ATGCTTGAGCAGCTTGAGAGTATAATCAACTTTGAAAAACTGACTGAATGGACCAGTggGGATCCACCGGTCatggcctcctccgaggacgtcatcaaggagttcatgcgcttcaaggtgcgcatggagggctccgtgaacggccacgagttcgagatcgagggcgagggcgagggccgcccctacgagggcacccagaccgccaagctgaaggtgaccaagggcggccccctgcccttcgcctgggacatcctgtcccctcagttccagtacggctccaaggcctacgtgaagcaccccgccgacatccccgactacttgaagctgtccttccccgagggcttcaagtgggagcgcgtgatgaacttcgaggacggcggcgtggtgaccgtgacccaggactcctccctgcaggacggcgagttcatctacaaggtgaagctgcgcggcaccaacttcccctccgacggccccgtaatgcagaagaagaccatgggctgggaggcctccaccgagcggatgtaccccgaggacggcgccctgaagggcgagatcaagatgaggctgaagctgaaggacggcggccactacgacgccgaggtcaagaccacctacatggccaagaagcccgtgcagctgcccggcgcctacaagaccgacatcaagctggacatcacctcccacaacgaggactacaccatcgtggaacagtacgagcgcgccgagggccgccactccaccggcgcctaa |
| ORF of B16 antigens | ATGTGTGCCGTGGGCGCCCTGGAGGGCCCTAGAAACCAGGATTGGCTGGGCGTGCCTAGGCAGCTGGGCGGATCCGGAGGCGGAGGATCCGGAGGATGCACAGCCCCCGATAACCTGGGCTACATGGGCGGCTCCGGCGGCGGAGGATCTGGAGGATGCTCCGTGTACGATTTCTTCGTGTGGCTGGGCGGCTCCGGAGGCGGTGGATCTGGAGGTCACAGCGGCCTGGTGACATTCCAGGCCTTCATCGACGTGATGTCCAGAGAGACCACCGACACAGATACAGCCGACGGCGGCAGCGGCGGCGGAGGCAGCGGAGGAAGCCCTGATGAGGTGGCCCTGGTGGAGGGCGTGCAGTCCCTGGGATTCACATACCTGAGACTGAAGGACAACTACGGCGGCTCCGGGGGCGGCGGATCTGGAGGCAGCAAGCCTAGCTTCCAGGAGTTCGTGGATTGGGAGAACGTGTCCCCTGAGCTGAACAGCACCGACCAGCCCTTCGGCGGCTCCGGTGGAGGAGGAAGCGGCGGAGGAACCGCCTTCTTCATCAACTTCATCGCCATCTACCACCACGCCAGCAGAGCCATCCCCTTCGGCACAATGGTGGGCGGCTCCGGCGGAGGAGGCTCTGGAGGAGTGGATAGAAACCCTCAGTTCCTGGACCCCGTGCTGGCCTACCTGATGAAGGGCCTGTGTGAGAAGCCCCTGGCCGGCGGAAGCCTGGGAGGAGGAGGATCCGGCGACTACAAGGACCACGACGGCGACTACAAAGACCACGATATCGACTACAAGGATGACGATGACAAGTGA |
| ORF of LL2 antigens and luciferase | ATGagccacagctccaaccttacccttcattaccgaactcTcctggtggaccggccctatgactgtaagtgtgggaaaggcggcagcggcggcggcggcagcggcggcaccaaaaccgaactggaactggcgctgagcccgattcattatagcagcgcgattccggcggcgggcagcaaccaggtgaccggcggcagcggcggcggcggcagcggcggcctgagcccgcgccattattatagcggctatagcagcagcctggaatatagcagcgaaagcacccataaaatttgggaacgcggGGATCCACCGGTCATGGAAGACGCCAAAAACATAAAGAAAGGCCCGGCGCCATTCTATCCGCTGGAAGATGGAACCGCTGGAGAGCAACTGCATAAGGCTATGAAGAGATACGCCCTGGTTCCTGGAACAATTGCTTTTACAGATGCACATATCGAGGTGGACATCACTTACGCTGAGTACTTCGAAATGTCCGTTCGGTTGGCAGAAGCTATGAAACGATATGGGCTGAATACAAATCACAGAATCGTCGTATGCAGTGAAAACTCTCTTCAATTCTTTATGCCGGTGTTGGGCGCGTTATTTATCGGAGTTGCAGTTGCGCCCGCGAACGACATTTATAATGAACGTGAATTGCTCAACAGTATGGGCATTTCGCAGCCTACCGTGGTGTTCGTTTCCAAAAAGGGGTTGCAAAAAATTTTGAACGTGCAAAAAAAGCTCCCAATCATCCAAAAAATTATTATCATGGATTCTAAAACGGATTACCAGGGATTTCAGTCGATGTACACGTTCGTCACATCTCATCTACCTCCCGGTTTTAATGAATACGATTTTGTGCCAGAGTCCTTCGATAGGGACAAGACAATTGCACTGATCATGAACTCCTCTGGATCTACTGGTCTGCCTAAAGGTGTCGCTCTGCCTCATAGAACTGCCTGCGTGAGATTCTCGCATGCCAGAGATCCTATTTTTGGCAATCAAATCATTCCGGATACTGCGATTTTAAGTGTTGTTCCATTCCATCACGGTTTTGGAATGTTTACTACACTCGGATATTTGATATGTGGATTTCGAGTCGTCTTAATGTATAGATTTGAAGAAGAGCTGTTTCTGAGGAGCCTTCAGGATTACAAGATTCAAAGTGCGCTGCTGGTGCCAACCCTATTCTCCTTCTTCGCCAAAAGCACTCTGATTGACAAATACGATTTATCTAATTTACACGAAATTGCTTCTGGTGGCGCTCCCCTCTCTAAGGAAGTCGGGGAAGCGGTTGCCAAGAGGTTCCATCTGCCAGGTATCAGGCAAGGATATGGGCTCACTGAGACTACATCAGCTATTCTGATTACACCCGAGGGGGATGATAAACCGGGCGCGGTCGGTAAAGTTGTTCCATTTTTTGAAGCGAAGGTTGTGGATCTGGATACCGGGAAAACGCTGGGCGTTAATCAAAGAGGCGAACTGTGTGTGAGAGGTCCTATGATTATGTCCGGTTATGTAAACAATCCGGAAGCGACCAACGCCTTGATTGACAAGGATGGATGGCTACATTCTGGAGACATAGCTTACTGGGACGAAGACGAACACTTCTTCATCGTTGACCGCCTGAAGTCTCTGATTAAGTACAAAGGCTATCAGGTGGCTCCCGCTGAATTGGAATCCATCTTGCTCCAACACCCCAACATCTTCGACGCAGGTGTCGCAGGTCTTCCCGACGATGACGCCGGTGAACTTCCCGCCGCCGTTGTTGTTTTGGAGCACGGAAAGACGATGACGGAAAAAGAGATCGTGGATTACGTCGCCAGTCAAGTAACAACCGCGAAAAAGTTGCGCGGAGGAGTTGTGTTTGTGGACGAAGTACCGAAAGGTCTTACCGGAAAACTCGACGCAAGAAAAATCAGAGAGATCCTCATAAAGGCCAAGAAGGGCGGAAAGATCGCCGTGTGA |
| ORF of exo-cellular domain of human EGFR | atgcttctcctggtgacaagccttctgctctgtgagttaccacacccagcattcctcctgatcccaGAGCAGAAACTCATCTCTGAAGAGGATCTGGGAGGCGGCGGTTCTCGCAAAGTGTGTAACGGAATAGGTATTGGTGAATTTAAAGACTCACTCTCCATAAATGCTACGAATATTAAACACTTCAAAAACTGCACCTCCATCAGTGGCGATCTCCACATCCTGCCGGTGGCATTTAGGGGTGACTCCTTCACACATACTCCTCCTCTGGATCCACAGGAACTGGATATTCTGAAAACCGTAAAGGAAATCACAGGGTTTTTGCTGATTCAGGCTTGGCCTGAAAACAGGACGGACCTCCATGCCTTTGAGAACCTAGAAATCATACGCGGCAGGACCAAGCAACATGGTCAGTTTTCTCTTGCAGTCGTCAGCCTGAACATAACATCCTTGGGATTACGCTCCCTCAAGGAGATAAGTGATGGAGATGTGATAATTTCAGGAAACAAAAATTTGTGCTATGCAAATACAATAAACTGGAAAAAACTGTTTGGGACCTCCGGTCAGAAAACCAAAATTATAAGCAACAGAGGTGAAAACAGCTGCAAGGCCACAGGCCAGGTCTGCCATGCCTTGTGCTCCCCCGAGGGCTGCTGGGGCCCGGAGCCCAGGGACTGCGTCTCTTGCCGGAATGTCAGCCGAGGCAGGGAATGCGTGGACAAGTGCAACCTTCTGGAGGGTGAGCCAAGGGAGTTTGTGGAGAACTCTGAGTGCATACAGTGCCACCCAGAGTGCCTGCCTCAGGCCATGAACATCACCTGCACAGGACGGGGACCAGACAACTGTATCCAGTGTGCCCACTACATTGACGGCCCCCACTGCGTCAAGACCTGCCCGGCAGGAGTCATGGGAGAAAACAACACCCTGGTCTGGAAGTACGCAGACGCCGGCCATGTGTGCCACCTGTGCCATCCAAACTGCACCTACGGATGCACTGGGCCAGGTCTTGAAGGCTGTCCAACGAATGGGCCTAAGATCCCGTCCATCGCCACTGGGATGGTGGGGGCCCTCCTCTTGCTGCTGGTGGTGGCCCTGGGGATCGGCCTCTTCATGCGACACCACCACCACCACCACTAA |
| ORF of anti-EGFR CAR construct | atgGCCTCACCGTTGACCCGCTTTCTGTCGCTGAACCTGCTGCTGCTGGGTGAGTCGATTATCCTGGGGAGTGGAGAAGCTGAGCAGAAACTCATCTCTGAAGAGGATCTGGGATCTGACATCCTGCTGACCCAGAGCCCCGTGATCCTGAGCGTGAGCCCCGGCGAGAGGGTGAGCTTCAGCTGCAGGGCCAGCCAGAGCATCGGCACCAACATCCACTGGTACCAGCAGAGGACCAACGGCAGCCCCAGGCTGCTGATCAAGTACGCCAGCGAGAGCATCAGCGGCATCCCCAGCAGGTTCAGCGGCAGCGGCAGCGGCACCGACTTCACCCTGAGCATCAACAGCGTGGAGAGCGAGGACATCGCCGACTACTACTGCCAGCAGAACAACAACTGGCCCACCACCTTCGGCGCCGGCACCAAGCTGGAGCTGAAGAGGACCGTGGCCGCCCCCAGCGTGTTCATCTTCCCCCCCAGCGACGAGCAGCTGAAGAGCGGCACCGCCAGCGTGGTGTGCCTGCTGAACAACTTCTACCCCAGGGAGGCCAAGGTGCAGTGGAAGGTGGACAACGCCCTGCAGAGCGGCAACAGCCAGGAGAGCGTGACCGAGCAGGACAGCAAGGACAGCACCTACAGCCTGAGCAGCACCCTGACCCTGAGCAAGGCCGACTACGAGAAGCACAAGGTGTACGCCTGCGAGGTGACCCACCAGGGCCTGAGCAGCCCCGTGACCAAGAGCTTCAACAGGGGCGCCGGCAGCACCAGCGGCAGCGGCAAGCCCGGCAGCGGCGAGGGCAGCACCAAGGGCCAGGTGCAGCTGAAGCAGAGCGGCCCCGGCCTGGTGCAGCCCAGCCAGAGCCTGAGCATCACCTGCACCGTGAGCGGCTTCAGCCTGACCAACTACGGCGTGCACTGGGTGAGGCAGAGCCCCGGCAAGGGCCTGGAGTGGCTGGGCGTGATCTGGAGCGGCGGCAACACCGACTACAACACCCCCTTCACCAGCAGGCTGAGCATCAACAAGGACAACAGCAAGAGCCAGGTGTTCTTCAAGATGAACAGCCTGCAGAGCAACGACACCGCCATCTACTACTGCGCCAGGGCCCTGACCTACTACGACTACGAGTTCGCCTACTGGGGCCAGGGCACCCTGGTGACCGTGAGCGCCGCCAGCACCAAGGGCCCCAGCGTGTTCCCCCTGGCCCCCAGCAGCAAGAGCACCAGCGGCGGCACCGCCGCCCTGGGCTGCCTGGTGAAGGACTACTTCCCCGAGCCCGTGACCGTGAGCTGGAACAGCGGCGCCCTGACCAGCGGCGTGCACACCTTCCCCGCCGTGCTGCAGAGCAGCGGCCTGTACAGCCTGAGCAGCGTGGTGACCGTGCCCAGCAGCAGCCTGGGCACCCAGACCTACATCTGCAACGTGAACCACAAGCCCAGCAACACCAAGGTGGACAAGAGGGTGGAGCCCAAGAGCATCGAaTTCATGTACCCCCCTCCCTACCTGGACAACGAGAGAAGCAACGGCACCATCATCCACATCAAAGAAAAGCACCTGTGCCACACCCAGAGCAGCCCCAAGCTGTTCTGGGCCCTGGTGGTGGTGGCCGGCGTGCTGTTCTGTTACGGCCTGCTGGTCACAGTGGCCCTGTGCGTGATCTGGACCAACAGCAGAAGAAACAGAGGCGGCCAGAGCGACTACATGAACATGACCCCCAGAAGGCCAGGCCTGACCAGAAAGCCCTACCAGCCCTACGCCCCTGCCAGAGACTTCGCCGCCTACAGACCCAGAGCCAAGTTCAGCAGATCCGCCGAGACAGCCGCCAACCTGCAGGATCCCAACCAGCTGTttAACGAGCTGAACCTGGGCAGACGGGAGGAATttGACGTGCTGGAAAAGAAGAGAGCCAGGGACCCCGAGATGGGCGGCAAGCAGCAGAGAAGAAGAAACCCTCAGGAAGGCGTCTACAACGCCCTGCAGAAAGACAAGATGGCCGAGGCCTACAGCGAGATCGGCACCAAGGGCGAGAGAAGAAGGGGCAAGGGCCACGATGGCCTGTttCAGGGCCTGTCCACCGCCACCAAGGACACCTttGACGCCCTGCACATGCAGACCCTGGCCCCCAGATGAgctcgagtctagaggatcaattccgcccccccccctaacgttactggccgaagccgcttggaataaggccggtgtgcgtttgtctatatgttattttccaccatattgccgtcttttggcaatgtgagggcccggaaacctggccctgtcttcttgacgagcattcctaggggtctttcccctctcgccaaaggaatgcaaggtctgttgaatgtcgtgaaggaagcagttcctctggaagcttcttgaagacaaacaacgtctgtagcgaccctttgcaggcagcggaaccccccacctggcgacaggtgcctctgcggccaaaagccacgtgtataagatacacctgcaaaggcggcacaaccccagtgccacgttgtgagttggatagttgtggaaagagtcaaatggctctcctcaagcgtattcaacaaggggctgaaggatgcccagaaggtaccccattgtatgggatctgatctggggcctcggtgcacatgctttacatgtgtttagtcgaggttaaaaaaacgtctaggccccccgaaccacggggacgtggttttcctttgaaaaacacgatgataatatggccacaaccatggcctcctccgaggacgtcatcaaggagttcatgcgcttcaaggtgcgcatggagggctccgtgaacggccacgagttcgagatcgagggcgagggcgagggccgcccctacgagggcacccagaccgccaagctgaaggtgaccaagggcggccccctgcccttcgcctgggacatcctgtcccctcagttccagtacggctccaaggcctacgtgaagcaccccgccgacatccccgactacttgaagctgtccttccccgagggcttcaagtgggagcgcgtgatgaacttcgaggacggcggcgtggtgaccgtgacccaggactcctccctgcaggacggcgagttcatctacaaggtgaagctgcgcggcaccaacttcccctccgacggccccgtaatgcagaagaagaccatgggctgggaggcctccaccgagcggatgtaccccgaggacggcgccctgaagggcgagatcaagatgaggctgaagctgaaggacggcggccactacgacgccgaggtcaagaccacctacatggccaagaagcccgtgcagctgcccggcgcctacaagaccgacatcaagctggacatcacctcccacaacgaggactacaccatcgtggaacagtacgagcgcgccgagggccgccactccaccggcgcctaa |
| ORF of D2GFP | atggtgagcaagggcgaggagctgttcaccggggtggtgcccatcctggtcgagctggacggcgacgtaaacggccacaagttcagcgtgtccggcgagggcgagggcgatgccacctacggcaagctgaccctgaagttcatctgcaccaccggcaagctgcccgtgccctggcccaccctcgtgaccaccctgacctacggcgtgcagtgcttcagccgctaccccgaccacatgaagcagcacgacttcttcaagtccgccatgcccgaaggctacgtccaggagcgcaccatcttcttcaaggacgacggcaactacaagacccgcgccgaggtgaagttcgagggcgacaccctggtgaaccgcatcgagctgaagggcatcgacttcaaggaggacggcaacatcctggggcacaagctggagtacaactacaacagccacaacgtctatatcatggccgacaagcagaagaacggcatcaaggtgaacttcaagatccgccacaacatcgaggacggcagcgtgcagctcgccgaccactaccagcagaacacccccatcggcgacggccccgtgctgctgcccgacaaccactacctgagcacccagtccgccctgagcaaagaccccaacgagaagcgcgatcacatggtcctgctggagttcgtgaccgccgccgggatcactctcggcatggacgagctgtacaagaagcttagccatggcttcccgccggaggtggaggagcaggatgatggcacgctgcccatgtcttgtgcccaggagagcgggatggaccgtcaccctgcagcctgtgcttctgctaggatcaatgtgtag |
| 5’UTR of mRNA construct | AAATAAGAGAGAAAAGAAGAGTAAGAAGAAATATAAGAGCCACC |
| 3’UTR of mRNA construct | GCTGCCTTCTGCGGGGCTTGCCTTCTGGCCATGCCCTTCTTCTCTCCCTTGCACCTGTACCTCTTGGTCTTTGAATAAAGCCTGAGTAGGAAGT |

**Table S2.**

Overview of the RNA vaccine used in the study

| Name | Descirption |
| --- | --- |
| CircRNA^D2GFP^ | A D2GFP-coding circRNA for analysis of protein expression in HEK293T cell lines. |
| mRNA^D2GFP^ | A D2GFP-coding m1Ψ-modified mRNA for analysis of protein expression in HEK293T cell lines. |
| CircRNA^luciferase^ | A lucifease-coding circRNA for analysis of protein expression in vivo. |
| CircRNA^SIINFEKL-RFP^ | A SIINFEKL antigen and RFP-coding circRNA for analysis of immune response and anti-tumor efficiency in vivo. |
| CircRNA^B16^ | A B16 endogenous antigen-coding circRNA for analysis of immune response and anti-tumor efficiency in B16 tumor model. |
| CircRNA^LL/2^ | A LL/2 endogenous antigen-coding circRNA for analysis of immune response and anti-tumor efficiency in LL/2 tumor model. |
| CircRNA^RFP^ | A RFP-coding circRNA as control RNA in the anti-tumor experiments |
| CircRNA^SIINFEKL-luc^ | A SIINFEKL antigen and luciferase-coding circRNA in the coculture assay with sorted APCs and antigen-specific T cells. |
| CircRNA^EGFR^ | A EGFR antigen-coding circRNA in the cocuture assay with DC2.4 cells and CAR-T cells. |
